# Exploring the potential benefits of stratified false discovery rates for region-based testing of association with rare genetic variation

**DOI:** 10.3389/fgene.2014.00011

**Published:** 2014-01-29

**Authors:** ChangJiang Xu, Antonio Ciampi, Celia M. T. Greenwood, The UK10K Consortium

**Affiliations:** ^1^Lady Davis Institute for Medical Research, Jewish General HospitalMontreal, QC, Canada; ^2^Department of Epidemiology, Biostatistics and Occupational Health, McGill UniversityMontreal, QC, Canada; ^3^Departments of Oncology and Human Genetics, McGill UniversityMontreal, QC, Canada

**Keywords:** rare genetic variants, SNV, false discovery rate, multiple testing, genomic annotation, whole genome sequencing, window-based tests, stratified false discovery rate

## Abstract

When analyzing the data that arises from exome or whole-genome sequencing studies, window-based tests, (i.e., tests that jointly analyze all genetic data in a small genomic region), are very popular. However, power is known to be quite low for finding associations with phenotypes using these tests, and therefore a variety of analytic strategies may be employed to potentially improve power. Using sequencing data of all of chromosome 3 from an interim release of data on 2432 individuals from the UK10K project, we simulated phenotypes associated with rare genetic variation, and used the results to explore the window-based test power. We asked two specific questions: firstly, whether there could be substantial benefits associated with incorporating information from external annotation on the genetic variants, and secondly whether the false discovery rate (FDRs) would be a useful metric for assessing significance. Although, as expected, there are benefits to using additional information (such as annotation) when it is associated with causality, we confirmed the general pattern of low sensitivity and power for window-based tests. For our chosen example, even when power is high to detect some of the associations, many of the regions containing causal variants are not detectable, despite using lax significance thresholds and optimal analytic methods. Furthermore, our estimated FDR values tended to be much smaller than the true FDRs. Long-range correlations between variants—due to linkage disequilibrium—likely explain some of this bias. A more sophisticated approach to using the annotation information may improve power, however, many causal variants of realistic effect sizes may simply be undetectable, at least with this sample size. Perhaps annotation information could assist in distinguishing windows containing causal variants from windows that are merely correlated with causal variants.

## Introduction

In genome-wide association studies, stringent testing thresholds have been recommended (and required by editors) to control the rate of identification of single nucleotide polymorphisms (SNPs) that may be falsely associated with a disease or trait of interest (Risch and Merikangas, 1996; Dudbridge and Gusnanto, 2008). The most commonly used threshold is 5 × 10^−8^, which controls the probability of making at least one false positive conclusion [the family-wise error rate (FWER)] to 5%, assuming approximately one million independent tests. Several studies have estimated approximately the same threshold, but derived it from different arguments: Risch and Merikangas used an early estimate of the potential number of genes in the genome (Risch and Merikangas, 1996), Dudbridge and Gusnanto examined the number of independent tests when performing infinitely dense genotyping of genetic polymorphisms (Dudbridge and Gusnanto, 2008), and empirical thresholds have been obtained using extensive permutation analyses (Li and Ji, 2005; Dudbridge and Gusnanto, 2008; Pe'er et al., 2008). This threshold assumes that all available common genetic polymorphisms are each tested against the disease, once. However, sequencing is now known to identify millions of genetic alterations that may be extremely rare—unique to one individual or only occurring in a small handful of people. These new sequence variants (single nucleotide variants or SNVs) may not have been previously observed, even in large collections of individuals such as the 1000 genomes (Abecasis et al., 2012), and linkage disequilibrium between these rare alterations and nearby known markers is usually very small. Previous estimates of genome-wide significance thresholds have not considered this spectrum of allele frequencies in their calculations (Xu et al., accepted), and as a result, the former standard significance threshold for controlling type 1 error rates may not be adequate (Browning and Thompson, 2012).

Using univariate tests, power to detect associations between SNVs and disease can be extremely low for rare SNVs, even when power is excellent for common variants. Hence, a whole new suite of test statistics has recently been developed to jointly analyze all of the genetic variation (in particular the rare genetic variation) in a chosen window or region of the genome (Asimit and Zeggini, 2010; Bansal et al., 2011; Burkett and Greenwood, 2013). One very popular method is the sequence kernel association test (SKAT) (Wu et al., 2011), which is a score test derived from a random effects model where the effects of all variants in a window are assumed to follow a normal distribution.

One challenge of these methods is that a single SNV may participate in several different tests resulting from a variety of different window choices, weighting strategies, or test statistics. Unlike SNP genotyping of common polymorphisms, where it is possible to estimate an upper bound on the number of SNPs to be tested, given known population-specific linkage disequilibrium patterns, the potential number of region-based tests performed has no known upper bound. Many different windows could be defined spanning the same genomic region (e.g., Brisbin et al., 2012). Furthermore, the genetic variants to be jointly analyzed do not need to be physically adjacent, but could, for example, lie in genes in the same pathway. Although choosing an appropriate threshold is already challenging when considering exome sequence data, the difficulties are exacerbated for whole-genome sequence data. In this latter context, window choices may be quite arbitrary.

Genome-wide significance thresholds for region-based tests do need to be established; we believe empirically-derived thresholds are probably necessary, and have recently shown that they can be effectively estimated by predicting the genome-wide threshold from empirical estimates obtained on smaller genomic regions (Xu et al., accepted). We demonstrated, in a sample of 2432 individuals from the UK10K consortium, that genome-wide thresholds for the SKAT test (Wu et al., 2011) in windows of 50 rare variants (overlapping by 25 variants) are expected to be near 7e-08.

Although it is certainly of interest to develop appropriate thresholds for deciding genome-wide significance for region-based tests, an alternative perspective on multiple testing corrections for sequence-based analyses may be worth exploring. Additional sources of repeated testing arise from the choice of which variants to prioritize and which test statistic to use. The range of different test statistics available can lead to very different results on the same set of variants; some statistics are most powerful when a large proportion of variants in a window are associated with an increasing disease risk (acting in the same direction), whereas other statistics may be optimal for smaller proportions of causal variants (Lee et al., 2012). Various minor allele frequency (MAF) thresholds can also be applied to restrict analysis to only “rare” genetic variants (Price et al., 2010). Also, scores such as PolyPhen-2 (Polymorphism Phenotyping v2) (Adzhubei et al., 2010) and SIFT (Sorting Intolerant From Tolerant) (Ng and Henikoff, 2002), which predict the probable functional impact of amino-acid changes induced by SNVs within exons, have been incorporated into region-based rare-variant tests. These publicly available genomic annotations can be used to select subsets of SNVs that are more likely to be associated with disease, or to give some SNVs more weight (Price et al., 2010; Wu et al., 2011; Lopes et al., 2012). Annotation of the entire genome is rapidly improving and non-coding regions are also known to contain many functional elements (Maher, 2012).

Therefore, it may not be sufficient to establish one significance threshold that will control the genome-wide FWER for region-based testing of rare genetic variation. An alternative strategy should be more open to exploiting external knowledge when deciding which associations are interesting, and these alternative approaches may lead to better power to detect true causal associations.

For a given choice of test statistic, windows, and weighting or prioritization of variants, permutation analysis of all the data will, of course, lead to an appropriate empirical family-wise significance threshold for any desired type 1 error rate. However, this is likely to require very large amounts of computation for genome-wide sequence data. Another possible approach to interpreting the results of multiple tests is to consider the false discovery rate (FDR) (Benjamini and Hochberg, 1995) instead of the FWER for choosing a significance threshold. The FWER is the probability of making at least one false rejection of the null hypothesis. For m independent tests, and if the probability of type 1 error for each test is chosen to be α, then the FWER rate can be written as FWER = 1 − (1 −α)^*m*^. Therefore, as the number of tests, *m*, increases, the significance threshold needed at each test, α, must get smaller in order to control FWER. In contrast, the FDR is the proportion of all rejected tests that are truly null—instead of controlling the probability of at least one false positive test, the FDR attempts to control the proportion of false positive tests which is a less stringent criterion.

FDR has several potential advantages over *p*-value measures of significance. Firstly, by controlling the (estimated) proportion of false positive associations among the total number of significant tests, it is usually possible to identify more true positive associations overall. This feature can be extremely beneficial to sensitivity and power when there is an apriori expectation of many true associations. Secondly, if the number of tests performed is increasing due to a range of choices for windows, test statistics, or variant selection in the same genomic regions, such tests can be expected to be positively correlated. An upper bound on the FDR has been demonstrated for situations with positive dependence (Benjamini and Yekutieli, 2001), such as would be expected in this situation. Furthermore, empirical estimates of FDR have been previously shown to be quite tolerant of correlations (Efron, 2007); in that paper by Efron, the estimated FDR rates were close to true values despite the correlations. However, recent work has highlighted that FDR estimates may be strongly biased and have very high variance in the presence of certain correlation patterns (Schwartzman and Lin, 2011). We therefore feel that the potential advantages of FDR methods over FWER methods in the presence of correlated tests warrant examination in the context of rare variant analysis.

Additionally, the use of stratified FDRs may enable further increases in power to detect associations. A stratified FDR analysis implies that the test statistics can be divided into subsets or strata with varying probability that the null hypothesis is true (Sun et al., 2006; Greenwood et al., 2007). Strata should be defined based on information that is external to the current study: for example, biologic plausibility or previously identified associations could be used to define strata. Estimates of FDR within each stratum can then be combined, and if there truly is variability in the proportion of null tests across strata, the sensitivity and specificity to detect true associations can be enhanced through the stratified analysis.

### Rationale

We therefore undertook this investigation to examine the power of window-based association tests, and how power is affected by analytic approaches that depend on external information such as genomic annotation. Due to the novelty of these window-based tests as well as the number of possible ways to implement them, analysts are likely to be particularly tempted to run multiple analyses with different choices for weights or subsets. Hence, we specifically wanted to know whether FDR estimation could be a beneficial approach to controlling the number of false-positive associations while simultaneously increasing power.

### Objectives

Based on our rationale, our goal is to answer the following questions:

- For window-based tests of rare genetic variation, can important power gains be achieved by considering external annotation information, either by weighting or by implementing a stratified FDR approach?
- Can FDR be accurately estimated for window-based tests of rare genetic variation?

To answer these questions, genetic sequencing data from a preliminary release of the UK10K project (www.uk10k.org), together with external information on amino acid alterations are the foundation for a simulation study comparing analytic strategies and their performance.

## Methods

### Data sets

Our analyses are based on simulated phenotypes together with genetic sequencing data from the UK10K project.

#### Sequencing data

The UK10K project is undertaking whole genome sequencing and analysis of approximately 10,000 individuals from the UK with the goal of understanding the contribution of rare genetic variation to common traits and diseases (www.uk10k.org). For region-based analysis of rare variants in this consortium, an initial analysis plan defined regions so that they contain 50 rare variants, where “rare” is either MAF <0.01 or MAF <0.05. Adjacent regions were chosen to overlap by 25 rare variants. To study correlation patterns, we used a portion of this sequencing data, specifically chromosome 3 sequencing data from an interim release, including 2432 individuals and 2,577,674 genetic variants. For an MAF threshold of 0.01 we defined 74,156 analysis windows or regions, derived from 1,853,923 rare variants at this threshold.

PolyPhen-2 scores, which predict the impact of amino acid changes on protein structure and function, were obtained for each exonic variant (Adzhubei et al., 2010). Among SNVs with MAF <0.01 on chromosome 3 in the UK10K data, there were 3296 variants with PolyPhen-2 scores indicating a “benign” alteration, 1219 variants coded as “possibly damaging,” and 2419 coded as “probably damaging.”

#### Simulated phenotype data—design

In order to achieve our objective of studying the performance of FDR estimation, it was necessary to implement a two-level simulation design. At the first level, we randomly selected a set of genes (and variants within genes) to influence the phenotype, and then using these causal variants, we simulated a continuous phenotype value for each individual in our data set. This step allowed us to calculate 74,156 region-based tests of association across chromosome 3, and thus obtain one estimate of FDR. Therefore the second level of the simulation design repeats this process 100 times, so that we can describe the variability in the FDR estimates and in the distribution of *p*-values. In addition, we repeated the entire process with three models varying the strength of the genetic influences.

#### Simulated phenotype data—details

Within each simulation, we randomly selected 40 genes to be causally related to the phenotype from the 1116 genes on the chromosome. Rare variants in and near these genes were then randomly sampled to be the influential genetic variants. We defined, for the purposes of selecting causal variants associated with each gene, all variants in the adjacent intergenic intervals as potentially associated with the selected gene. We excluded singleton variants from consideration since they provide little additional benefit to power in tests of rare genetic variation (Ladouceur et al., 2013).

The risk of a variant being causal, and its effect on a continuous phenotype, were generated as a function of the PolyPhen-2 score values. The phenotypes *y*_*i*_, *i* = 1, …, *n*, for *n* individuals, were simulated from the model *y*_*i*_ = ∑_*j*_ β_*j*_ *x*_*ij*_ + ϵ_*i*_ where ϵ_*i*_ ~ *N*(0, σ) represents random variability in the *i*th person's phenotype. The genotype covariates, *x*_*ij*_, represent the number of minor alleles for individual *i* at causal variant *j*, and the effects of the causal variants, β_*j*_, were randomly generated following the parameters in Table 1. Three different values were chosen for the standard deviation associated with the random variability, σ ∈ (0.5, 1.0, 1.5).

**Table 1:** **Parameter values for simulating phenotypes, dependent on PolyPhen-2 scores**.

**PolyPhen-2 score**	**Probability that variant is causal**	**Mean of Normally distributed effect on phenotype**	**Standard deviation normally distributed effect on phenotype**
Benign	0.05	1.00	0.5
Possibly damaging	0.35	1.65	0.5
Probably damaging	0.45	2.00	0.5
Missing	0.001	1.20	0.5

Background random variability in phsenotype was normally generated with mean 0 and standard deviation between 0.5 and 1.5.

For each value of σ, 100 sets of causal genes and variants were randomly selected, and correspondingly, 100 different phenotypes were generated for each person.

### Data analyses

In general, the analysis plan involved region-based tests of association for all defined windows on chromosome 3, followed by examination of the joint distribution of the resulting *p*-values in order to estimate sensitivity and FDR. These two phases were each undertaken using several strategies to account for external annotation information about the genetic variants.

#### Region-based tests of association using SKAT

For each set of phenotypes across all individuals, we performed region-based SKAT tests of association (Wu et al., 2011) between the generated phenotype set and all 74,156 partially-overlapping regions of 50 rare variants (MAF <0.01) on chromosome 3. SKAT is designed to test association between a set of genetic variants, such as those identified by sequencing methods in a small genomic window, and a phenotype or trait. The regions or windows for analysis must be chosen by the analyst, and may reflect gene boundaries or may be simply an arbitrary partitioning of the data on each chromosome. For a continuous phenotype, the SKAT test statistic can be written

$$Q=(y-\hat{\mu })\prime K(y-\hat{\mu }),$$

where *y* is a vector of phenotype values, $\hat{\mu }$ is the predicted mean of *y* under null hypothesis, and *K* is the SKAT kernel matrix, which depends on the genotype matrix and a choice of variant weights. Under the null hypothesis, the distribution of *Q* is asymptotically equal to a positive quadratic form of standard normal distributions, and the *p*-value can be calculated using Davies exact method or other approximation methods (Wu et al., 2011).

#### Implementation of SKAT analyses on chromosome 3, incorporating annotation information

The SKAT window-based analyses were performed in three different ways to give different priority to variants with genomic annotations.

1. Analysis type “N”: We used the default SKAT weighting of variants which ignores any annotation information and we included only variants with MAF <0.01. Here the weights were defined to give more weight to variants with smaller minor allele frequencies following a Beta distribution with parameters *a*_1_ = 1 and *a*_2_ = 25 (Wu et al., 2011).
2. Analysis type “P”: Here we weighted each variant also by PolyPhen-2 scores. For this purpose, we assigned weights of 0.5 for unknown or missing PolyPhen-2 scores, 0.25 for benign variation, 0.75 for possibly damaging, and 0.85 for probably damaging scores. Note, this analysis assumed that variants known to involve benign changes to the protein were given lower weights than variants where the change had unknown impact.
3. Analysis type “S”: Here we analyzed only the subset of rare variants in a window that had non-missing PolyPhen-2 scores. That is, variants were included in the analysis if they had been assessed as benign, possibly damaging or probably damaging. Note that the window boundaries were not changed, so for this strategy there could be very few variants jointly analyzed in a window, and many windows (especially those outside genes) contained no annotated variants and were not analyzed with strategy S. The default SKAT weighting of variants was also used here.

#### Defining strata and analysis strategies for FDR estimation

After analysis, region-based test results were summarized across all windows (All), windows that contained no variants with damaging PolyPhen-2 scores (Stratum 1), and windows that contained at least one variant with a PolyPhen-2 score of probably or possibly damaging (Stratum 2). Note, therefore, that analysis type “S” of Stratum 2 (S-Stratum2) describes windows containing only damaging variants (possibly or probably), S-Stratum1 describes windows containing non-annotated or benign variants, and that “S-All” are the windows with any kind of variants. Furthermore, we categorized windows into those truly containing at least one causal variant (H1), and windows where at least one variant was strongly correlated with a causal SNP at either *r* = 0.9 (H1-Corr0.90) or *r* ≥ 0.75 (H1-Corr0.75). All the nomenclature is presented in Table 2. By considering the strata, as well as the treatment of annotated variants within the test statistics, many analysis strategies were defined. For example, “S-Stratum2” is the analysis of only the damaging variants (and the windows in which they occur); whereas “N-All” implies an analysis of all windows and all variants, with default SKAT weights.

**Table 2:** **Nomenclature used to describe combinations of analytic strategies and subsets of regions analyzed**.

**Description**	**Nomenclature**
SKAT tests without any weighting based on PolyPhen-2 scores, all rare variants with MAF <0.01	N
SKAT tests with weighting based on PolyPhen-2 scores, all rare variants with MAF <0.01	P
SKAT tests only on rare variants (MAF <0.01) with benign, possibly damaging, or probably damaging PolyPhen-2 scores	S
All windows	All
Windows that contained only variants with either missing or benign PolyPhen2 scores	Stratum 1 or St1
Windows that contained at least one variant with a possibly or probably damaging PolyPhen-2 score	Stratum 2 or St2
A stratified analysis that combines results from Stratum 1 and Stratum 2	Strat or Str
Windows containing at least one causal variant	H1
Windows containing at least one variant correlated with a causal SNP at *r* ≥ 0.9	H1-Corr0.90
Windows containing at least one variant correlated with a causal SNP at *r* ≥ 0.75	H1-Corr0.75
Standard error associated with random error or noise	σ

#### “true” sensitivity and FDR

Within the set of tests arising from each analytic strategy, we calculated the true sensitivity and the true value of the FDR for several chosen *p*-value thresholds. Let *p*_*w*_ represent the *p*-value for window *w* using a particular testing strategy, and let α be a *p-value* threshold defining whether the null hypothesis is rejected. With some abuse of notation, let *H*1_*w*_ represent the logical event that the window *w* truly contains at least one causal variant. We can therefore define “true” sensitivity as

$$\text{tSENS}=\frac{\sum_{w\;=\;1}^{W}\left[I(H1_{w})\cap I\left(p_{w}<\alpha \right)\right]}{\sum_{w\;=\;1}^{W}I(H1)},$$

where the sum is across all *w* = 1, … *W* windows tested. Similarly the “true” FDR can be written

$$\text{tFDR}=\;\frac{\sum_{W=\text{1}}^{w}\left[I\left(p_{w}<\alpha \right)\cap I\left({}^{\circ}H1_{w}\right)\right]}{\sum_{w=1}^{W}I\left(p_{w}<\alpha \right)},$$

where *I*(° *H*1_*w*_) indicates that a window does not contain a causal variant. These sensitivities and FDRs were averaged across the 100 simulations for each threshold α.

#### Estimation of FDR

We estimated FDRs using three different methods, and compared the estimates to the true values. The three FDR estimation methods that we used are the Benjamini and Hochberg (BH) correction of *p*-values (Benjamini and Hochberg, 1995), the beta-uniform model-based parametric approach (BUM) (Pounds and Morris, 2003), and the modified Grenander density estimator based semi-pararametric approach (software called fdrtool) (Strimmer, 2008), in each case we estimate the tail area-based FDR. The BH method is a step-up *p*-value adjustment which controls FDR at level τ. Let all the *p*-values for a given testing strategy be ordered from smallest to largest, *p*_(1)_, *p*_(2)_, …, *p*_(*w*)_, …, *p*_(*W*)_. The Benjamini–Hochberg procedure finds w^*^, the largest value of *w*, such that $p_{\left(w^{*}\right)}\leq \frac{w^{*}}{W}\tau$, and rejects the null hypothesis for all tests with *p*-values smaller than or equal to *p*_(w^*^)_. Pounds and Morris (2003) use the fact that *p*-values under the null hypothesis are expected to follow a uniform distribution, and therefore, they assume that the distribution of all *p*-values follows a mixture distribution where the uniform is mixed with a Beta distribution, denoted *f*_1_,

$$f(p)=\pi +\left(1-\pi \right)f_{1}(p).$$

Here, π represents the proportion of test statistics that follow the null hypothesis. The estimated FDR for *p*-value threshold α is then the proportion of rejected tests estimated to follow the uniform distribution, and can be written

$${\text{FDR}}_{\text{BUM}}(\alpha )=\frac{{\hat{\pi }}_{ub}\alpha }{\hat{F}(\alpha )-\left(1-\alpha \right){\hat{\pi }}_{ub}+{\hat{\pi }}_{ub}\alpha }$$

Where ${\hat{\pi }}_{ub}$ represents an estimated upper bound on π, and $\hat{F}$(.) is the estimated mixture distribution. Instead of assuming a uniform distribution for *p*-values under the null hypothesis, the method of Strimmer (2008) finds an empirical null distribution by using a smoothing approach. The mixture distribution is written more generally as

$$f(p)=\eta_{0}f_{0}(p)+\left(1-\eta_{0}\right)f_{1}(p).$$

Strimmer writes FDR at a given *p*-value *p*_*i*_ as

$${\text{FDR}}_{\text{S}}(p_{i})=\eta_{0}\frac{1-F_{0}(p_{i};\;\theta )}{1-F(p_{i})}$$

where *F*(.) and *F*_0_(.) represent the distribution functions for the mixture model and the null distribution, respectively. A Grenander density estimator is used to obtain a nonparametric estimate of the distribution *F* and a truncated maximum likelihood is used to estimate the null density. Therefore, these three methods encompass very different approaches to the estimation of FDRs.

An approach conceptually similar to Strimmer's was taken by Efron (Efron and Tibshirani, 1998; Efron, 2007) and implemented in the program locfdr (http://cran.r-project.org/web/packages/locfdr/index.html), but an empirical smoother of the histogram of test statistics was used instead of the Grenander function. We were unable to obtain reasonable results with this method and they are not shown.

#### Stratified FDR estimation

To implement stratified FDR estimation, the FDR was estimated separately in each stratum. For the combined analysis, a desired FDR threshold was chosen and applied separately to the results for each stratum. This induces different *p*-value cutoffs and different sensitivity rates across the strata, which are then combined to obtain overall estimates of sensitivity and power.

#### Single point analyses

For comparison, results from a small number of simulations were analyzed using single-marker tests of association. Each SNV along chromosome 3 was tested for association with the phenotype, using an additive (0, 1, 2) coding for the number of minor alleles at the genetic variant.

## Results

### Simulated phenotype data

For each simulation, the phenotypes were designed to depend on genotypes at a randomly selected set of 40 genes. Then within the chosen set of genes, a variable number of causal variants were sampled. Table 3 displays the mean number of causal variants selected by our two-stage phenotype simulation design, both for all variants and among the subset of variants with PolyPhen-2 scores. It is evident from Table 3 that almost 50% of the causal variants had PolyPhen-2 annotations, and therefore that, as desired, the proportion of annotated variants that are causal is much higher than the proportion of non-annotated causal variants. Also the number of windows that contained at least one causal variant is very equally split between Stratum 1 and 2. Table 3 also shows that only a small number of analysis windows contained at least one causal variant. However, the number of windows that contain variants strongly correlated with a causal SNV is much higher. It is worth noting that although 40 genes were chosen to influence phenotype, it is possible that no causal variants were randomly selected for some of these genes. In fact the number of genes containing at least one causal variant varied between 8 and 22 across the simulations.

**Table 3:** **Number of causal variants generated in the simulations**.

**Category**	**Number of causal variants**	**Number of windows containing at least one causal variant**	**Number of windows containing at least one variant correlated with a causal variant at**	
			*****r*** ≥ **0.9****	*****r*** ≥ **0.75****
All variants	21.89 (4.57)	41.02 (8.26)	1174.78 (1037.12)	1709.08 (1442.99)
Variants in Stratum 1	10.61 (3.18)	20.96 (6.30)	443.65 (726.19)	779.94 (1106.35)
Variants in Stratum 2	11.28 (2.75)	20.96 (4.89)	758.79 (786.22)	970.75 (995.43)
Variants with PolyPhen-2 scores	12.46 (3.12)	22.81 (5.25)	845.97 (826.83)	1190.50 (1110.22)

Each simulation first selected 40 genes and then selected variants to be causal in and/or near these genes. Mean (and standard deviation) are reported across the simulations.

### Region-based tests of association: distributions of *p*-values

**Figure 1:**
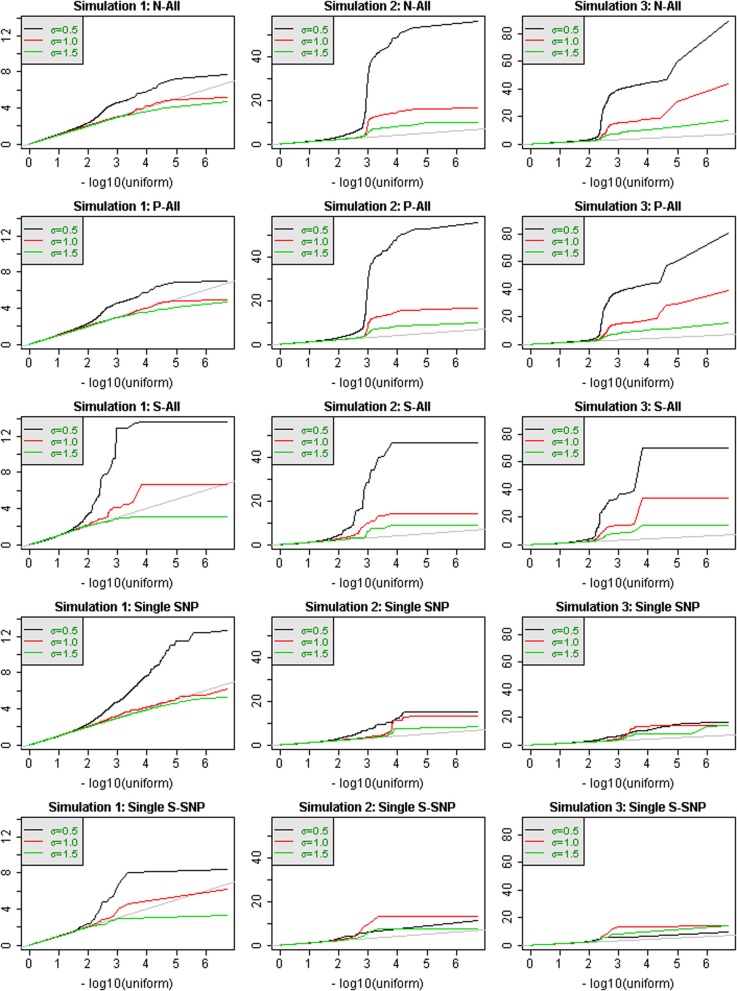
**QQ-plots of *p*-values for analysis of all chromosome 3 windows, for 3 different simulated phenotypes that were selected to illustrate performance differences**. Values of σ are distinguished by color. Region-based testing using three analysis strategies (N-All, P-All, and S-All) are shown in the first three rows. The bottom two rows show the results of single-SNP analyses, either with all SNPS (row 4) or only annotated SNPs (row 5).

Figure 1 shows QQ-plots of *p*-values for 3 simulations. The 3 columns in Figure 1 correspond to 3 different sets of simulated phenotypes, and the rows correspond to different analytic strategies. Region-based test results (rows 1–3) are contrasted with single-SNP tests (rows 4–5). When σ = 0.5, the power to detect association at the window level can be extremely substantial and the QQ-plots may deviate markedly from the line of expectation. For example, the smallest *p*-values for simulation #3 reach 10^−80^. It is also notable that the most significant test statistics can vary by almost an order of magnitude across different simulations. In contrast, when σ = 1.5, there is very little power to detect association with the genetic variation in the windows, at a sample size of 2432 individuals, for any method. Single SNP results can have more power than region-based tests, as in simulation #1, or less power, as in simulations 2 and 3.

**Figure 2:**
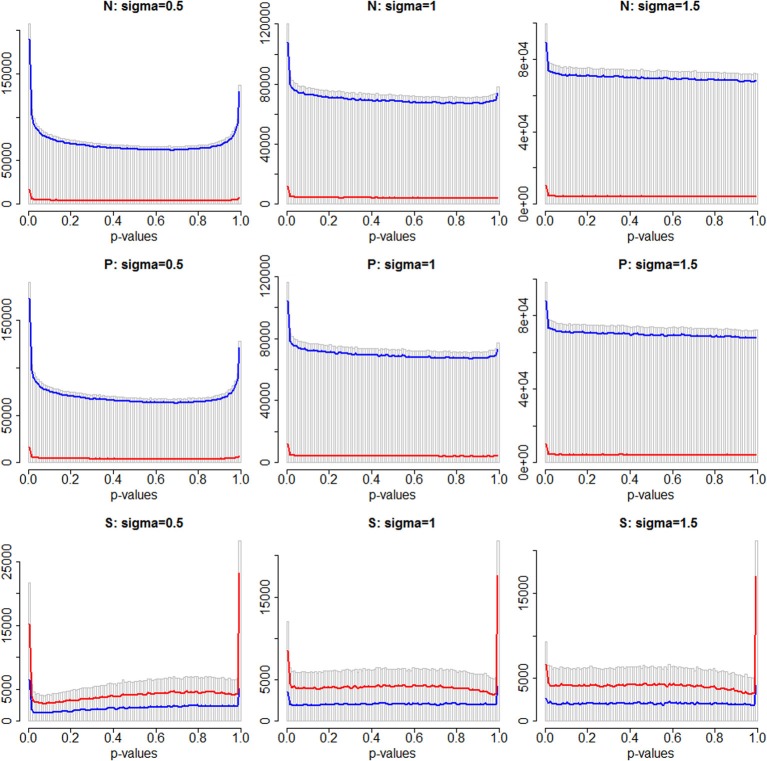
**Distributions of *p*-values across 100 simulations, for window-based tests of variation at all windows on chromosome 3 (gray histogram), for Stratum 1 (blue), and Stratum 2 (red)**. The first row shows the standard SKAT weighting of variants (N), the second row shows analyses weighted by PolyPhen-2 scores (P), and the bottom row shows analyses of only the subset of damaging variants (S). Columns 1 to 3 are σ = 0.5, 1.0 and 1.5, respectively.

Figure 2 shows histograms of the *p*-values obtained for all region-based tests of association for three different simulations, for all tests, and also separately for Stratum 1 (no damaging causal variants in the windows) and Stratum 2 (at least one damaging causal variant in the window). In each histogram there is a visible peak of small *p*-values, indicating that a subset of the tests deviate markedly from the null hypothesis but there is also an apparent peak of *p*-values at or near the value of 1.0. This latter spike is more visible when the background random variability is at its smallest (σ = 0.5), and particularly dramatic for the “S” analytic strategy where only annotated variants were analyzed. Therefore, we believe that this spike is likely due to violations of the asymptotic convergence of the test statistics. For the S strategy, the data sets of analyzed variants could be very sparse—a much smaller number of windows were analyzed, and within those windows there may have been little genetic variability. Since there was a small number of damaging variants, a window may contain only one or two analyzable variants. Furthermore, only a few individuals may carry alternative alleles due to the rarity of the high risk alleles. When σ = 0.5, individuals carrying one of these causal variants would appear to have extreme phenotypes (large outliers) and this may compromise the convergence of the test statistics.

The tests in Stratum 2 are a very small minority of all tests for the N and P analysis strategies, but a large proportion of all tests for the S strategy. This can be seen in Figure 2 in the bottom row, where the line representing Stratum 2 is higher than Stratum 1. Visually, it is not very easy to distinguish, in any row of this figure, whether one stratum contains a higher proportion of small *p*-values than the other.

### True sensitivity and FDR for different analysis strategies

**Figure 3:**
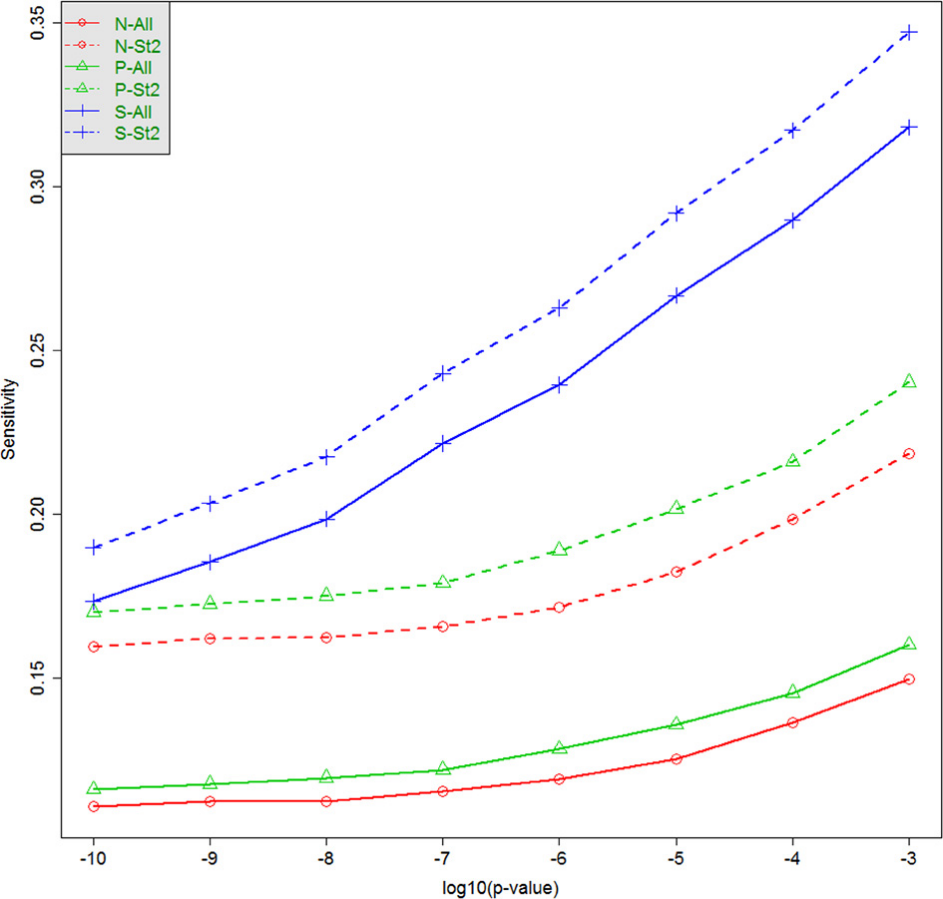
**Sensitivity (tSENS) at different significance thresholds (*p*-values) and for various analytic strategies**. The horizontal axis is −log_10_ (*p*), and the vertical axis is the true sensitivity for detecting windows containing at least one associated causal variant, when σ = 0.5. N-All: All tests, standard weighting; N-St2: Stratum 2, standard weighting; P-All: All tests, PolyPhen-2 weights; P-St2: Stratum 2, PolyPhen-2 weights; S-All: All tests; subset of damaging variants; S-St2: Stratum 2, subset of damaging variants.

**Figure 4:**
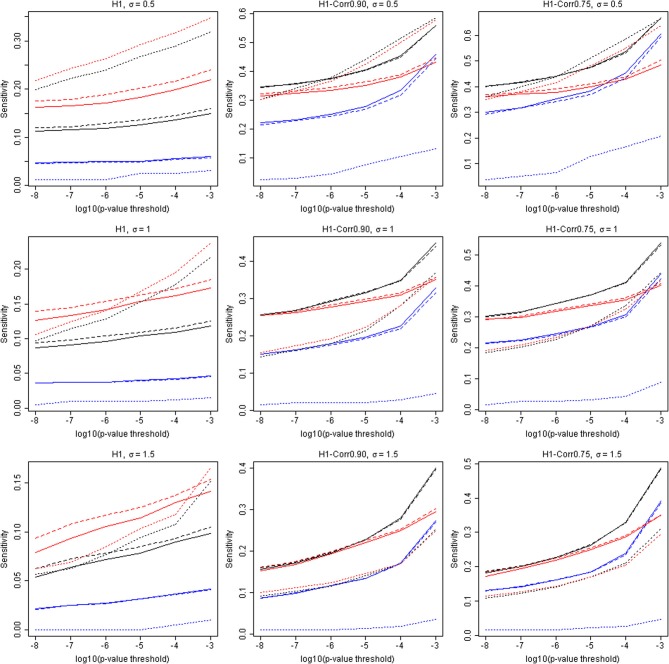
**Additional results for sensitivities (tSENS) and the *p*-value threshold**. The horizontal axis is −log_10_(*p*), and the vertical axis is the true sensitivity for detecting windows containing at least one associated causal variant. Solid lines: Analytic strategy “N”. Dashed lines: Analytic strategy “P”. Dotted lines: Analytic strategy “S.” Three different colors represent All-tests (black), Stratum 1 (blue), and Stratum 2 (red), respectively. The three rows show results for σ = 0.5, 1.0, and 1.5, respectively. The three columns contain results for H1, H1-Corr0.90, and H1-Corr0.75, respectively.

The capacity to identify windows containing causal variants is shown in Table 4 and Figure 3. For a given *p*-value threshold for significance, the sensitivity is the proportion, of the windows where the null hypothesis was rejected, that actually contain at least one true causal variant. For a *p*-value threshold of 1e-05, the performance of five different analytic strategies are presented in Table 4. Firstly all windows are analyzed with the default SKAT weights based on MAF (N–All). We then separate windows containing benign or non-annotated variants from windows containing at least one damaging variant and analyzed these two sets separately with standard SKAT (N–Stratum1 and N–Stratum 2). For Stratum 2 only, we also report analyses either with weights (P-Stratum 2) or restricting to the subset of annotated variants (S-Stratum 2) using PolyPhen-2 weights (P–Stratum 2). Results are also shown for all windows, for windows containing at least one causal variant, and for windows correlated with a causal window (see Table 2 for nomenclature details). Figure 3 contains some additional sensitivity results for Stratum 1 tests for σ = 0.5. For complete results, see Tables S1A–D, 3A–D as well as Figure 4.

**Table 4:** **Sensitivity (tSENS) for detection of windows containing causal variants with different analysis strategies, for a *p*-value threshold of 1e-05**.

**Analysis strategy**		**Sensitivity (tSENS), i.e., the proportion of causal windows identified**			**Proportion of windows where null is falsely rejected, the tFDR**		
	σ	**Windows: H1**	**Windows: H1-Corr0.90**	**Windows: H1-Corr0.75**	**Windows:H1**	**Windows: H1-Corr0.90**	**Windows: H1-Corr0.75**
N—All	0.5	0.13 (0.10)	0.40 (0.15)	0.48 (0.16)	0.98 (0.03)	0.92 (0.08)	0.90 (0.09)
N—All	1.5	0.08 (0.08)	0.23 (0.17)	0.26 (0.20)	0.90 (0.24)	0.83 (0.25)	0.81 (0.25)
N—Stratum 1	0.5	0.05 (0.10)	0.28 (0.18)	0.38 (0.22)	0.997 (0.006)	0.97 (0.03)	0.96 (0.04)
N—Stratum 1	1.5	0.03 (0.07)	0.13 (0.16)	0.18 (0.21)	0.94 (0.24)	0.91 (0.24)	0.90 (0.24)
N—Stratum 2	0.5	0.18 (0.14)	0.35 (0.19)	0.40 (0.20)	0.88 (0.16)	0.83 (0.16)	0.82 (0.16)
N—Stratum 2	1.5	0.11 (0.10)	0.22 (0.18)	0.25 (0.19)	0.71 (0.37)	0.65 (0.35)	0.64 (0.34)
P—Stratum 2	0.5	0.20 (0.14)	0.36 (0.19)	0.41 (0.20)	0.86 (0.17)	0.81 (0.17)	0.80 (0.17)
P—Stratum 2	1.5	0.13 (0.11)	0.23 (0.18)	0.25 (0.20)	0.69 (0.37)	0.65 (0.35)	0.64 (0.34)
S—Stratum 2	0.5	0.30 (0.14)	0.43 (0.14)	0.48 (0.14)	0.81 (0.10)	0.75 (0.10)	0.72 (0.09)
S—Stratum 2	1.5	0.10 (0.10)	0.15 (0.12)	0.17 (0.13)	0.61 (0.35)	0.56 (0.33)	0.53 (0.31)

Mean (standard deviation) across 100 simulations. The proportion of windows where the null is falsely rejected is the “true” FDR value.

Examining strategy H1, where the focus is only on windows containing causal variants, it can be seen that the sensitivities tend to be very low for any analytic strategy, and they do not increase much as the *p*-value threshold becomes less stringent. This conclusion appears to contradict the highly deviant QQ-plot results seen in Figure 1, in particular when σ = 0.5. It appears, therefore, that some windows containing true causal variants can be detected with ease (with high power), but many others appear to be indistinguishable from the windows containing no causal variants. The best sensitivity for windows truly containing at least one causal variant (H1), with a liberal *p*-value threshold of 1e-3 and for the S-Stratum 2 analytic strategy, reaches only 35%.

However, when we relax our definition of sensitivity, the results improve. Our more inclusive definition calls a “causal window” as a window containing at least one variant in strong linkage disequilibrium with a causal variant, where linkage disequilibrium is defined using *r* ≥ 0.9 (H1-Corr0.90) or *r* ≥ 0.75 (H1-Corr0.75). Sensitivity then measures the proportion of this larger set of windows with *p*-values below the chosen threshold. Table 4 shows that sensitivity increases substantially for all analytic strategies, and dramatically for N-All and Stratum 1. Using this liberal definition of a causal window based on correlation of 0.75, sensitivity reaches 48% with σ = 0.5, a *p*-value threshold of 1e-05, and either N-All or S-Stratum2 (Table 4).

The “true” FDR, or the proportion of rejected tests where the window contained no causal variants, is also shown in Table 4. It is evident from the very high tFDR values that most of the significant tests correspond to windows that did not contain any causal variants, even when we relax or enlarge our definition of success to include windows that are strongly correlated with those containing causal variants. More focused subsets of windows, obtained for example by the S-Stratum2 analyses, have smaller tFDR estimates, but they are still greater than 50% even when a *p-value* of 1e-08 is used as the threshold (Table S3A). These results demonstrate clearly that it is extremely difficult to segregate causal and non-causal windows.

### Estimation of FDR

Using three methods for estimating FDR, we then examined the proportion of falsely-rejected hypotheses across methods, analytic strategies, and with the true values. Table 5 shows, of the tests with FDR estimates less than 0.05 (i.e., an FDR threshold of 0.05), what proportion is truly null. Tables S2A–I, S4A–I show complete results for different analytic strategies and three different FDR thresholds.

**Table 5:** **The performance of estimated FDR methods for window-based rare variant tests, using three different methods for estimation**.

**Analytic strategy**	**Identification of windows containing causal variants**			**Identification of windows either containing causal variants, or correlated with such windows at *r* ≥ 0.90**		
	**Benjamini–Hochberg (BH)**	**Beta-uniform mixture (BUM)**	**FDRtool**	**Benjamini–Hochberg (BH)**	**Beta-uniform mixture (BUM)**	**FDRtool**
N—All	0.981 (0.033)	0.982 (0.030)	0.982 (0.030)	0.935 (0.080)	0.925 (0.088)	0.936 (0.079)
N—Strat	0.980 (0.036)	0.980 (0.042)	0.980 (0.036)	0.952 (0.049)	0.944 (0.056)	0.953 (0.049)
P—All	0.974 (0.064)	0.970 (0.107)	0.975 (0.060)	0.925 (0.096)	0.911 (0.130)	0.927 (0.091)
P—Strat	0.971 (0.075)	0.970 (0.073)	0.972 (0.075)	0.943 (0.078)	0.932 (0.087)	0.943 (0.078)
S—All	0.927 (0.038)	0.927 (0.037)	0.920 (0.040)	0.874 (0.046)	0.858 (0.046)	0.862 (0.045)
S—Strat	0.927 (0.037)	0.926 (0.038)	0.920 (0.040)	0.882 (0.045)	0.868 (0.045)	0.872 (0.046)

Results show the proportion of windows containing no causal variants (tFDR) for an estimated FDR of 0.05. Results are shown for the simulation with σ = 0.5. Results are the mean across 100 simulations (standard deviation).

Focusing on only windows that truly contain causal variants (H1), Table 5 indicates that over 90% of the rejected tests are actually null across a variety of analytic strategies, in stark contrast to the estimated FDR values of 0.05. All three methods give very similar results. In fact, the three methods identify the same windows as significant: the true null proportions are very similar across the methods.

By expanding the definition of a “true” association to include windows that are correlated with causal windows, there is some improvement. The proportion of null rejections drops from well over 90% to as low as 40–50% when using the BUM method, and in the Stratum 2 subset analyses, but these values are still far higher than the estimated FDR of 0.05. When using a slightly more liberal definition of a true association (*r* ≥ 0.75; Tables S4A–I) the proportion of null rejection falls further. When examining other FDR thresholds (Tables S2/4B, S2/4E, S2/4H show FDR = 0.25; Tables S2/4C, S2/4F, S2/4I show FDR = 0.50) the estimated FDR values are even closer to 1.0.

### Stratified FDR

A stratified analysis strategy allows for different *p*-value thresholds to be applied in different strata. This reflects variability in the estimated proportions of truly null hypotheses across strata. The results of stratified FDR analysis are also shown in Table 5 and in the Supplementary Tables. For a chosen FDR threshold (here FDR = 0.05), different *p*-value thresholds are applied in the two strata. However, we see no benefit in terms of the number of falsely-associated windows or sensitivities associated with the stratified analysis.

## Discussion

In this paper, we have explored the potential of using FDRs together with genomic annotation to improve the ability to detect associations with rare genetic variants using window-based tests. Returning to our Objectives, we found that, as expected, using annotation information improved power, since this was built into our simulation design. However, power remained low. Also, we did not find that FDR estimation was a particularly useful tool in this context. The proportion of significant yet not-associated windows was very large, and the estimates of FDR were extremely biased. We discuss this bias below.

We based our exploration on an interim release of sequencing data from the UK10K project, including approximately 2.5 million sequence-identified variants on chromosome 3 among 2432 individuals, and implemented a fairly complex simulation design. We assumed that there were as many as 40 genes on this chromosome with influence on a continuous trait. We randomly selected 40 genes from those on the chromosome, and then we randomly selected causal genetic variants from all genetic variants in or near these genes with probabilities that depended on the real PolyPhen-2 scores for the genetic variation. As an alternate strategy, we could have fixed the genes and variants selected to be causal, and simply varied their effect on phenotype across the simulations. However, our chosen approach incorporates additional variability in the pattern of associated variants and their correlations, since we wanted to examine the performance of FDR estimation under a variety of conditions. Also, for reasons of feasibility, we used pilot data on only one chromosome. Patterns of gene density and correlations may vary across chromosomes, but our simulation design hopefully includes enough randomness that results, in general, would be applicable to larger regions or different chromosomes.

The choice of as many as 40 causal genes located on the same chromosome was made for two reasons. Firstly, for many complex traits, it may be extremely likely that there are large numbers of genes each with a small influence on the trait; this has been suggested for height (Yang et al., 2010), for example, as well as several other continuous phenotypes. Secondly, in order to estimate FDRs using many of the existing methods, it is necessary to estimate the proportion of associated (non-null) tests. However, if this proportion is too close to zero, then estimation becomes extremely difficult. In fact, despite the choice of 40 causal genes, the number of windows containing a causal variant is still small (Table 3).

Although the number of causal variants is small, the number of windows potentially showing association could be much larger due to patterns of linkage disequilibrium leading to extensive correlations. We therefore implemented a more relaxed definition of successfully identifying a true signal: if a window showing a significant result contained at least one genetic variant strongly correlated with a causal variant (using either *r* ≥ 0.90 or *r* ≥ 0.75), then we counted this as a true identification. Sensitivity increased quite substantially with the relaxed definition, and in some models reached 50–60%. If we had used a lower level of correlation when defining a “true positive” region identification, we would undoubtedly have been able to improve our sensitivity further. In fact, the correlation, *r*, is not ideal as a measure of the strength of linkage disequilibrium between variants, especially for rare genetic variants. A more nuanced consideration of haplotypic structure could provide an interesting perspective and may lead to improved sensitivities. Our relaxed definition of success also raises questions about how to perform fine mapping, since “true positive” windows could be quite genetically distant from any causal variants, if there was long-range disequilibrium. Of course, many studies of real phenotypes have identified associations that are located far from any likely gene.

One of our most striking findings was the discrepancy between estimated FDR values (e.g., FDR = 0.05) and the true FDR based on the proportion of windows with small *p*-values that either contain a causal variant or are strongly associated with causal variants. Three factors play into this, power, the proportion of all tests that are null (π), and the correlations between windows. Table 6 shows a standard 2 × 2 table setup for calculating sensitivity, specificity and false positive rates, where $\text{FDR}=\frac{C}{A\;+\;C}$. Poor power leads to values of *A* that are too small. Furthermore, if *C*+D is a very large number (π is large) then it becomes easy for *C* to be much larger than *A*. Finally, the linkage disequilibrium structure leads to complex patterns of dependence; signals resulting from causal genetic variants may be detected in windows some distance away. Hence, the choice of definition for a “causal” window influences the number of tests placed into the two columns.

**Table 6:** **Setup for calculation of sensitivity, power, and false positive rates**.

**Region-based test result**	**Truth**		
	**True H_*A*_**	**True H_0_**	**Total**
*p*-value < Threshold: reject null hypothesis	A	C	A + C
*p*-value ≥ Threshold: do not reject null hypothesis	B	D	B + D
Total	A + B	C + D	

A, B, C, D represent the numbers of tests that fall into the four cells defined by the p-value (whether or not the null hypothesis is rejected) and whether (H_A_) or not (H_0_) the region is a causal region.

The lack of power for detection of many of the causally-associated regions may have been due to very small MAFs, to the presence of only a very small number of causal variants in each window, or even to the fact that with 41 causal variants on average (Table 3), that the separate signals would be difficult to distinguish. Better power may be obtainable by iteratively correcting the phenotype for each consecutively-identified variant or signal, and re-running analyses on the residuals, although it would be tricky to decide exactly how to implement such a strategy for window-based testing.

If window-based tests perform poorly when there are only a few causal variants, perhaps single-SNP tests have more power. Therefore, in a few selected cases, we compared window-based results to SNP results (Figure 1), and in fact we concluded that there is not necessarily one strategy that will be more powerful. The relative performance will depend on the density of causal variants in small genomic regions, and whether several causal variants occur within the same window. This comparison is not the primary focus of this paper, but could be an interesting question for future research.

We compared three methods for estimation of FDRs. The BH method (Benjamini and Hochberg, 1995) implements a step-up strategy adjusting each *p*-value in turn, and provides an upper bound for the FDR under certain patterns of dependency (Benjamini and Yekutieli, 2001). This method does not require an estimate of the proportion of the tests that are truly null. In contrast, we implemented two methods that do estimate this proportion, one parametric (Pounds and Morris, 2003) and one semi-parametric (Strimmer, 2008). Despite the very different model assumptions, in fact the resulting estimates of FDR were very comparable across the three methods. However, none of the estimates were at all close to the true FDR values. All methods were probably being misled by associations seen at correlated variants, located in nearby windows. Performance for the model-based methods may also have been adversely affected by the observed spike in the number of *p*-values of 1.0 (Figure 2). The presence of this spike violates the assumption of a uniform distribution of *p*-values under the null hypothesis. Finally, it is plausible that the differences between the approaches would be more visible for smaller FDR estimates.

Annotation of the genome, including regulatory regions as well as genomic conservation, is improving daily (Maher, 2012). It seems intuitive that such information should aid in identifying associations between genetic variability and phenotypes. We simulated data where PolyPhen-2 scores influenced not only the probability that a variant was causal, but also the magnitude of the effect of the variant on the phenotype. It was therefore not surprising to find that use of weighted test statistics, where weights were derived from the PolyPhen-2 scores, improved the sensitivity and power. Similarly, analyses of only the subset of possibly- or probably-damaging annotated variants performed better than analysis of all variants, as expected. The differences in power, however, were quite small and smaller than we had previously anticipated. Although we could have prepared a more complex set of relationships between genomic annotation information and our simulated phenotypes, perhaps using multiple different annotation measures, we do not feel that our primary conclusions here would be altered, given the same analysis strategies. Power of the tests will still be largely driven by the MAFs and density of the causal variants within the windows, as well as the magnitude of their effects. An interesting point to consider here is that when small *p*-values are seen in correlated windows—that do not contain any causal variants but are in linkage disequilibrium with causal variants—the windows with the smallest *p*-values may be less likely to contain annotated variants due to MAF variation. Annotation information may therefore be useful for fine-mapping.

Another option for coping with multiple testing would be to develop a Bayesian model for the strength of each test statistic's association, where informative prior distributions are assumed for the parameters measuring the strength of association between genomic regions and phenotype. This approach could be considered conceptually as an extension of stratified FDR to the case where each test statistic has its own stratum, defined by the genomic annotation information for all variants in each region. The result of such an analysis would be a (posterior) probability that the genetic variation in a chosen region is associated with the phenotype. Regions with annotations likely to contain causal genetic variation would have higher prior probabilities of association with phenotype, and hence also higher posterior probabilities of association. This avenue may be worth further consideration and exploration.

Prior to undertaking this simulation study, our hypothesis was that use of stratified FDR methods, using genomic annotation information, could lead to improved power to detect associations with rare genetic variation. We did not find any improved performance using stratified FDR methods in this context. However in another context where the strata may more clearly delineate the probability of a true association, or if informative prior information could be constructed effectively, then perhaps a return to consideration of these issues would be warranted.

### Conflict of interest statement

The authors declare that the research was conducted in the absence of any commercial or financial relationships that could be construed as a potential conflict of interest.

## Supplementary material

Table S1 **(A–D)** True sensitivity (tSENS) and true FDR (tFDR) for different analytic strategies. Each table shows the tSENS and tFDR values for different *p*-value thresholds, ranging from 1e-08 (Table S1A) to 1e-03 (Table S1D). “m” is the mean over the 10 simulations, and “sd” is the standard deviation. Nomenclature follows Table 2.

Table S2 **(A–I)** Sensitivities and proportion of truly null window tests (tFDR) for different estimated values of FDR. Each table shows the sensitivity and tFDR values associated with a different estimated FDR threshold between 0.05 **(S2A)** and 0.50 **(S2I)**. Nomenclature follows Table 2.

Table S3 **(A–D)** True sensitivity (tSENS) and true FDR (tFDR) for different analytic strategies examining correlated windows with variants correlated at *r*≥ 0.75. Each table shows the tSENS and tFDR values for different *p*-value thresholds, ranging from 1e-08 (Table S1A) to 1e-03 (Table S1D). “m” is the mean over the 10 simulations, and “sd” is the standard deviation. Nomenclature follows Table 2.

Table S4 **(A–I)** Sensitivities and proportion of truly null window tests (tFDR) for different estimated values of FDR examining correlated windows with variants correlated at *r* ≥ 0.75. Each table shows the sensitivity and tFDR values associated with a different estimated FDR threshold between 0.05 **(S2A)** and 0.50 **(S2I)**. Nomenclature follows Table 2.
