# Corrigendum: Exploring the potential benefits of stratified false discovery rates for region-based testing of association with rare genetic variation

**DOI:** 10.3389/fgene.2014.00061

**Published:** 2014-03-26

**Authors:** Celia M. T. Greenwood

**Affiliations:** Lady Davis Institute for Medical Research, Jewish General HospitalMontreal, QC, Canada

**Keywords:** Acknowledgments, false discovery rates, rare genetic variation, UK10K, region-based tests

Regrettably, the Acknowledgments were omitted from the submitted manuscript and are therefore included here in this Corrigendum

