# Supplementary material for: Exploring the potential benefits of stratified false discovery rates for region-based testing of association with rare genetic variation
Source: Front Genet. 2014 Jan 29;5:11. doi: 10.3389/fgene.2014.00011 (PMC3905218; doi:10.3389/fgene.2014.00011)
Supplement: Table S1 — (A–D) True sensitivity (tSENS) and true FDR (tFDR) for different analytic strategies. Each table shows the tSENS and tFDR values for different p-value thresholds, ranging from 1e-08 (Table S1A) to 1e-03 (Table S1D). “m” is the mean over the 10 simulations, and “sd” is the standard deviation. Nomenclature follows Table 2. [file DataSheet1.ZIP › greenwood supp/10.3389.fgene.2014.00011 _Greenwood_Supplementary Table_1.PDF]

Tables S1.A - S1.D. True sensitivity (tSENS) and true FDR (tFDR) for different analytic strategies, H1-Corr≥ 0.90

Table S1.A. p-value threshold is 10^{-8}

	FDR				Sensitivity			
	H1		H1-Corr		H1		H1-Corr	
	m	sd	m	sd	m	sd	m.sen	sd.sen
N-All-σ0.5	0.9422	0.1706	0.8766	0.1833	0.1125	0.0957	0.3460	0.1751
N-St1-σ0.5	0.9557	0.1963	0.9308	0.1960	0.0468	0.0943	0.2220	0.1835
N-St2-σ0.5	0.8549	0.2323	0.8129	0.2246	0.1624	0.1371	0.3153	0.1952
N-All-σ1.0	0.8524	0.3058	0.7986	0.2977	0.0869	0.0808	0.2553	0.1697
N-St1-σ1.0	0.8824	0.3129	0.8627	0.3086	0.0364	0.0789	0.1511	0.1690
N-St2-σ1.0	0.7538	0.3334	0.6983	0.3187	0.1261	0.1142	0.2540	0.1736
N-All-σ1.5	0.7151	0.4072	0.6627	0.3915	0.0539	0.0578	0.1550	0.1529
N-St1-σ1.5	0.7140	0.4478	0.6984	0.4395	0.0214	0.0517	0.0867	0.1425
N-St2-σ1.5	0.6036	0.4181	0.5538	0.3966	0.0789	0.0854	0.1525	0.1471
P-All-σ0.5	0.9259	0.1950	0.8687	0.1959	0.1196	0.0942	0.3444	0.1712
P-St1-σ0.5	0.9556	0.1963	0.9298	0.1961	0.0464	0.0943	0.2150	0.1825
P-St2-σ0.5	0.8426	0.2247	0.8034	0.2163	0.1752	0.1372	0.3235	0.1935
P-All-σ1.0	0.8480	0.3053	0.7984	0.2979	0.0941	0.0843	0.2541	0.1709
P-St1-σ1.0	0.8815	0.3134	0.8590	0.3123	0.0364	0.0789	0.1513	0.1694
P-St2-σ1.0	0.7447	0.3376	0.6984	0.3193	0.1398	0.1257	0.2549	0.1739
P-All-σ1.5	0.7242	0.3967	0.6737	0.3808	0.0623	0.0624	0.1589	0.1544
P-St1-σ1.5	0.7241	0.4429	0.7078	0.4359	0.0211	0.0534	0.0860	0.1423
P-St2-σ1.5	0.6204	0.4056	0.5694	0.3873	0.0935	0.0940	0.1605	0.1486
S-All-σ0.5	0.7632	0.1585	0.6849	0.1607	0.1987	0.1249	0.3036	0.1466
S-St1-σ0.5	0.7468	0.4338	0.7430	0.4324	0.0125	0.0743	0.0258	0.1274
S-St2-σ0.5	0.7302	0.1694	0.6639	0.1702	0.2176	0.1354	0.3029	0.1534
S-All-σ1.0	0.6779	0.3270	0.6078	0.3154	0.0974	0.0906	0.1438	0.1125
S-St1-σ1.0	0.3460	0.4755	0.3435	0.4727	0.0050	0.0500	0.0150	0.1114
S-St2-σ1.0	0.6612	0.3304	0.5985	0.3174	0.1065	0.0987	0.1542	0.1218
S-All-σ1.5	0.5195	0.4156	0.4491	0.3765	0.0565	0.0701	0.0913	0.0899
S-St1-σ1.5	0.1700	0.3775	0.1675	0.3728	0.0000	0.0000	0.0100	0.1000
S-St2-σ1.5	0.5154	0.4139	0.4472	0.3751	0.0621	0.0771	0.0994	0.0982

Table S1.B. p-value threshold is 10^{-6}

	FDR				Sensitivity			
	H1		H1-Corr		H1		H1-Corr	
	m	sd	m	sd	m	sd	m.sen	sd.sen
N-All-σ0.5	0.9740	0.0354	0.9067	0.1068	0.1192	0.0964	0.3749	0.1689
N-St1-σ0.5	0.9864	0.0999	0.9562	0.1086	0.0500	0.0960	0.2527	0.1824
N-St2-σ0.5	0.8653	0.2022	0.8188	0.1961	0.1716	0.1366	0.3334	0.1934
N-All-σ1.0	0.8682	0.2941	0.8180	0.2814	0.0962	0.0865	0.2925	0.1802
N-St1-σ1.0	0.8947	0.3000	0.8730	0.2951	0.0377	0.0794	0.1779	0.1817
N-St2-σ1.0	0.7761	0.3205	0.7282	0.3054	0.1416	0.1270	0.2773	0.1816
N-All-σ1.5	0.7827	0.3659	0.7174	0.3535	0.0720	0.0725	0.1953	0.1705
N-St1-σ1.5	0.8254	0.3756	0.8007	0.3717	0.0273	0.0707	0.1170	0.1567
N-St2-σ1.5	0.6521	0.3972	0.6015	0.3775	0.1056	0.0990	0.1931	0.1652
P-All-σ0.5	0.9508	0.1411	0.8949	0.1472	0.1285	0.0953	0.3739	0.1676
P-St1-σ0.5	0.9763	0.1404	0.9482	0.1418	0.0483	0.0953	0.2450	0.1786
P-St2-σ0.5	0.8441	0.2225	0.7995	0.2149	0.1891	0.1395	0.3450	0.1899
P-All-σ1.0	0.8851	0.2662	0.8282	0.2604	0.1037	0.0883	0.2934	0.1780
P-St1-σ1.0	0.9047	0.2862	0.8826	0.2819	0.0377	0.0794	0.1741	0.1801
P-St2-σ1.0	0.7807	0.3078	0.7356	0.2942	0.1542	0.1309	0.2822	0.1837
P-All-σ1.5	0.7727	0.3677	0.7151	0.3554	0.0781	0.0757	0.1966	0.1707
P-St1-σ1.5	0.8254	0.3756	0.8008	0.3715	0.0266	0.0706	0.1171	0.1565
P-St2-σ1.5	0.6456	0.3964	0.6009	0.3770	0.1171	0.1067	0.1987	0.1697
S-All-σ0.5	0.8104	0.1168	0.7361	0.1234	0.2396	0.1307	0.3780	0.1481
S-St1-σ0.5	0.9375	0.2385	0.9265	0.2403	0.0125	0.0743	0.0444	0.1535
S-St2-σ0.5	0.7714	0.1271	0.7085	0.1327	0.2631	0.1403	0.3670	0.1462
S-All-σ1.0	0.6871	0.2826	0.6323	0.2779	0.1285	0.1077	0.1783	0.1303
S-St1-σ1.0	0.5143	0.4985	0.5118	0.4967	0.0100	0.0704	0.0200	0.1214
S-St2-σ1.0	0.6740	0.2821	0.6222	0.2772	0.1402	0.1182	0.1911	0.1424
S-All-σ1.5	0.5961	0.3816	0.5336	0.3621	0.0767	0.0774	0.1154	0.1025
S-St1-σ1.5	0.2200	0.4163	0.2175	0.4123	0.0000	0.0000	0.0100	0.1000
S-St2-σ1.5	0.5808	0.3813	0.5269	0.3633	0.0845	0.0852	0.1231	0.1093

Table S1.C. p-value threshold is 10^{-5}

	FDR				Sensitivity			
	H1		H1-Corr		H1		H1-Corr	
	m	sd	m	sd	m	sd	m.sen	sd.sen
N-All-σ0.5	0.9759	0.0312	0.9154	0.0831	0.1254	0.0958	0.4044	0.1522
N-St1-σ0.5	0.9970	0.0058	0.9713	0.0319	0.0507	0.0975	0.2790	0.1842
N-St2-σ0.5	0.8811	0.1592	0.8334	0.1573	0.1824	0.1362	0.3511	0.1914
N-All-σ1.0	0.9312	0.1941	0.8762	0.1930	0.1040	0.0887	0.3144	0.1797
N-St1-σ1.0	0.9552	0.1962	0.9307	0.1974	0.0401	0.0825	0.1948	0.1825
N-St2-σ1.0	0.7856	0.3096	0.7444	0.2954	0.1535	0.1322	0.2926	0.1850
N-All-σ1.5	0.8980	0.2394	0.8257	0.2492	0.0783	0.0762	0.2261	0.1733
N-St1-σ1.5	0.9350	0.2377	0.9091	0.2440	0.0314	0.0746	0.1339	0.1640
N-St2-σ1.5	0.7076	0.3738	0.6539	0.3533	0.1140	0.1044	0.2202	0.1760
P-All-σ0.5	0.9694	0.0445	0.9099	0.0843	0.1358	0.0961	0.4038	0.1536
P-St1-σ0.5	0.9968	0.0064	0.9696	0.0351	0.0493	0.0951	0.2699	0.1833
P-St2-σ0.5	0.8635	0.1724	0.8137	0.1683	0.2017	0.1403	0.3645	0.1877
P-All-σ1.0	0.9270	0.1858	0.8650	0.1911	0.1091	0.0895	0.3160	0.1772
P-St1-σ1.0	0.9553	0.1963	0.9257	0.2054	0.0391	0.0805	0.1923	0.1791
P-St2-σ1.0	0.8027	0.2882	0.7624	0.2767	0.1624	0.1336	0.2978	0.1853
P-All-σ1.5	0.9048	0.2223	0.8328	0.2411	0.0847	0.0802	0.2274	0.1735
P-St1-σ1.5	0.9449	0.2182	0.9186	0.2261	0.0314	0.0746	0.1339	0.1640
P-St2-σ1.5	0.6944	0.3737	0.6504	0.3530	0.1250	0.1120	0.2257	0.1814
S-All-σ0.5	0.8504	0.0811	0.7790	0.0834	0.2669	0.1299	0.4420	0.1442
S-St1-σ0.5	0.9857	0.1013	0.9713	0.1103	0.0262	0.1114	0.0757	0.1970
S-St2-σ0.5	0.8103	0.0963	0.7471	0.0967	0.2920	0.1384	0.4256	0.1396
S-All-σ1.0	0.7260	0.2322	0.6697	0.2355	0.1528	0.1124	0.2122	0.1340
S-St1-σ1.0	0.5767	0.4938	0.5753	0.4928	0.0100	0.0704	0.0200	0.1214
S-St2-σ1.0	0.7062	0.2337	0.6572	0.2364	0.1676	0.1231	0.2223	0.1474
S-All-σ1.5	0.6292	0.3447	0.5651	0.3265	0.0940	0.0856	0.1404	0.1125
S-St1-σ1.5	0.3200	0.4688	0.3155	0.4633	0.0000	0.0000	0.0133	0.1051
S-St2-σ1.5	0.6122	0.3456	0.5587	0.3295	0.1031	0.0933	0.1462	0.1170

Table S1.D. p-value threshold is 10^{-3}

	FDR				Sensitivity			
	H1		H1-Corr		H1		H1-Corr	
	m	sd	m	sd	m	sd	m.sen	sd.sen
N-All-σ0.5	0.9909	0.0060	0.9591	0.0278	0.1498	0.0993	0.5581	0.1207
N-St1-σ0.5	0.9986	0.0024	0.9836	0.0116	0.0603	0.1083	0.4578	0.1888
N-St2-σ0.5	0.9289	0.0638	0.8713	0.0833	0.2187	0.1398	0.4311	0.1678
N-All-σ1.0	0.9857	0.0117	0.9355	0.0339	0.1181	0.0962	0.4477	0.1328
N-St1-σ1.0	0.9976	0.0045	0.9770	0.0152	0.0466	0.0897	0.3276	0.1909
N-St2-σ1.0	0.9100	0.0969	0.8348	0.1287	0.1727	0.1385	0.3511	0.1755
N-All-σ1.5	0.9849	0.0139	0.9215	0.0395	0.0984	0.0885	0.3990	0.1287
N-St1-σ1.5	0.9970	0.0057	0.9723	0.0215	0.0418	0.0862	0.2729	0.1710
N-St2-σ1.5	0.9193	0.0921	0.8477	0.1229	0.1412	0.1232	0.2949	0.1866
P-All-σ0.5	0.9891	0.0068	0.9553	0.0299	0.1602	0.0969	0.5575	0.1197
P-St1-σ0.5	0.9985	0.0025	0.9825	0.0127	0.0578	0.1059	0.4452	0.1902
P-St2-σ0.5	0.9167	0.0718	0.8583	0.0985	0.2404	0.1407	0.4463	0.1663
P-All-σ1.0	0.9837	0.0130	0.9346	0.0341	0.1249	0.0983	0.4390	0.1324
P-St1-σ1.0	0.9975	0.0046	0.9771	0.0164	0.0459	0.0883	0.3142	0.1980
P-St2-σ1.0	0.8860	0.1458	0.8202	0.1598	0.1856	0.1450	0.3566	0.1757
P-All-σ1.5	0.9828	0.0147	0.9197	0.0411	0.1049	0.0897	0.3962	0.1253
P-St1-σ1.5	0.9970	0.0059	0.9720	0.0214	0.0408	0.0853	0.2694	0.1717
P-St2-σ1.5	0.8928	0.1393	0.8240	0.1562	0.1538	0.1288	0.3006	0.1846
S-All-σ0.5	0.9294	0.0334	0.8768	0.0401	0.3182	0.1398	0.5845	0.1333
S-St1-σ0.5	0.9977	0.0091	0.9872	0.0231	0.0312	0.1210	0.1318	0.2453
S-St2-σ0.5	0.9021	0.0462	0.8467	0.0521	0.3473	0.1452	0.5777	0.1425
S-All-σ1.0	0.8589	0.0852	0.7804	0.0839	0.2176	0.1224	0.3685	0.1220
S-St1-σ1.0	0.9630	0.1757	0.9548	0.1771	0.0147	0.0772	0.0449	0.1439
S-St2-σ1.0	0.8206	0.1064	0.7501	0.1043	0.2375	0.1319	0.3571	0.1333
S-All-σ1.5	0.8352	0.1371	0.7561	0.1399	0.1515	0.1201	0.2517	0.1530
S-St1-σ1.5	0.8871	0.3141	0.8803	0.3136	0.0100	0.0704	0.0350	0.1485
S-St2-σ1.5	0.8044	0.1519	0.7361	0.1590	0.1650	0.1311	0.2479	0.1618
