# Supplementary material for: Exploring the potential benefits of stratified false discovery rates for region-based testing of association with rare genetic variation
Source: Front Genet. 2014 Jan 29;5:11. doi: 10.3389/fgene.2014.00011 (PMC3905218; doi:10.3389/fgene.2014.00011)
Supplement: Table S1 — (A–D) True sensitivity (tSENS) and true FDR (tFDR) for different analytic strategies. Each table shows the tSENS and tFDR values for different p-value thresholds, ranging from 1e-08 (Table S1A) to 1e-03 (Table S1D). “m” is the mean over the 10 simulations, and “sd” is the standard deviation. Nomenclature follows Table 2. [file DataSheet1.ZIP › greenwood supp/10.3389.fgene.2014.00011 _Greenwood_Supplementary Table_2.PDF]

Tables S2.A - S2.I. Sensitivities and proportion of truly null window tests (tFDR) for different estimated values of FDR (H1-Corr≥ 0.90)

Table S2.A. The estimated FDR is 0.05 using the method: BH

	H1		H1-Corr	
	FDR	sensitivity	FDR	sensitivity
N-St1-σ0.5	0.9981 (0.0036)	0.0586 (0.112)	0.9763 (0.0296)	0.3653 (0.2287)
N-St2-σ0.5	0.9116 (0.1066)	0.2205 (0.1472)	0.8611 (0.1132)	0.4209 (0.1976)
N-All-σ0.5	0.9808 (0.0332)	0.1441 (0.1022)	0.9348 (0.0801)	0.4785 (0.1804)
N-Str-σ0.5	0.9797 (0.036)	0.1499 (0.1038)	0.952 (0.0493)	0.393 (0.1761)
N-St1-σ1.0	0.896 (0.3003)	0.0419 (0.0832)	0.8738 (0.2961)	0.2252 (0.2056)
N-St2-σ1.0	0.7982 (0.3062)	0.1696 (0.1381)	0.7605 (0.2936)	0.3237 (0.1968)
N-All-σ1.0	0.8753 (0.2949)	0.1101 (0.094)	0.8302 (0.2841)	0.3394 (0.1933)
N-Str-σ1.0	0.8745 (0.2946)	0.1143 (0.0943)	0.847 (0.2866)	0.2806 (0.1739)
N-St1-σ1.5	0.8357 (0.3667)	0.0371 (0.0855)	0.8167 (0.3644)	0.1492 (0.1833)
N-St2-σ1.5	0.7381 (0.36)	0.1377 (0.1197)	0.6926 (0.3436)	0.2694 (0.1942)
N-All-σ1.5	0.8107 (0.3512)	0.089 (0.0869)	0.7557 (0.3417)	0.2504 (0.1919)
N-Str-σ1.5	0.8331 (0.3238)	0.0944 (0.0872)	0.7949 (0.32)	0.2168 (0.1638)
P-St1-σ0.5	0.9879 (0.0999)	0.0567 (0.1058)	0.9652 (0.1021)	0.3571 (0.2244)
P-St2-σ0.5	0.8996 (0.1182)	0.2388 (0.1456)	0.8516 (0.1205)	0.4263 (0.195)
P-All-σ0.5	0.9735 (0.0641)	0.1513 (0.101)	0.9252 (0.0958)	0.4761 (0.1803)
P-Str-σ0.5	0.9712 (0.0745)	0.159 (0.0999)	0.9429 (0.078)	0.3927 (0.1736)
P-St1-σ1.0	0.8959 (0.3003)	0.0413 (0.0833)	0.8766 (0.2959)	0.213 (0.1952)
P-St2-σ1.0	0.8181 (0.2665)	0.1828 (0.1463)	0.7785 (0.2569)	0.3383 (0.1985)
P-All-σ1.0	0.8826 (0.2814)	0.1144 (0.0956)	0.8382 (0.2714)	0.3357 (0.1905)
P-Str-σ1.0	0.8974 (0.2439)	0.1215 (0.098)	0.8701 (0.2394)	0.2836 (0.174)
P-St1-σ1.5	0.8158 (0.3843)	0.0364 (0.0844)	0.8031 (0.3786)	0.1465 (0.1821)
P-St2-σ1.5	0.7341 (0.3576)	0.1464 (0.1239)	0.6906 (0.342)	0.2713 (0.1939)
P-All-σ1.5	0.7852 (0.3673)	0.0933 (0.0876)	0.7466 (0.3506)	0.2494 (0.192)
P-Str-σ1.5	0.8191 (0.3345)	0.0988 (0.0879)	0.7885 (0.3272)	0.2165 (0.1628)
S-St1-σ0.5	0.9977 (0.0091)	0.0312 (0.121)	0.9856 (0.0286)	0.1318 (0.2453)
S-St2-σ0.5	0.9014 (0.049)	0.3461 (0.1457)	0.8463 (0.0552)	0.5756 (0.1461)
S-All-σ0.5	0.9271 (0.0378)	0.3154 (0.141)	0.8735 (0.0456)	0.5794 (0.1356)
S-Str-σ0.5	0.9274 (0.0369)	0.3173 (0.1405)	0.8824 (0.0449)	0.5366 (0.1415)
S-St1-σ1.0	0.6864 (0.4629)	0.0125 (0.0743)	0.6801 (0.4602)	0.0312 (0.1321)
S-St2-σ1.0	0.7772 (0.1511)	0.2159 (0.1321)	0.7061 (0.1557)	0.3139 (0.1486)
S-All-σ1.0	0.7967 (0.159)	0.1933 (0.1177)	0.7198 (0.1615)	0.3045 (0.1492)
S-Str-σ1.0	0.8019 (0.15)	0.1968 (0.1219)	0.7365 (0.1549)	0.2861 (0.1369)
S-St1-σ1.5	0.4586 (0.4996)	0.005 (0.05)	0.4546 (0.4961)	0.0183 (0.1158)
S-St2-σ1.5	0.6643 (0.3306)	0.1218 (0.1105)	0.613 (0.3165)	0.1733 (0.139)
S-All-σ1.5	0.6723 (0.3325)	0.1104 (0.1005)	0.6061 (0.3145)	0.1726 (0.1352)
S-Str-σ1.5	0.6925 (0.3203)	0.1114 (0.1014)	0.6429 (0.3094)	0.1575 (0.1262)

Table S2.B. The estimated FDR is 0.25 using the method: BH

	H1		H1-Corr	
	FDR	sensitivity	FDR	sensitivity
N-St1-σ0.5	0.999 (0.0016)	0.0736 (0.1174)	0.9849 (0.0187)	0.5319 (0.2346)
N-St2-σ0.5	0.9455 (0.0623)	0.2636 (0.1524)	0.899 (0.0803)	0.5317 (0.2042)
N-All-σ0.5	0.9918 (0.0112)	0.168 (0.1156)	0.9615 (0.0484)	0.6104 (0.1701)
N-Str-σ0.5	0.9912 (0.0105)	0.1818 (0.1139)	0.9721 (0.0246)	0.529 (0.1855)
N-St1-σ1.0	0.9375 (0.2381)	0.0492 (0.0968)	0.9184 (0.2346)	0.3021 (0.2347)
N-St2-σ1.0	0.8502 (0.2601)	0.1877 (0.1409)	0.8058 (0.254)	0.3883 (0.219)
N-All-σ1.0	0.9233 (0.2356)	0.1193 (0.0998)	0.8794 (0.2317)	0.4151 (0.2101)
N-Str-σ1.0	0.9329 (0.2025)	0.1276 (0.1009)	0.9032 (0.2024)	0.349 (0.1973)
N-St1-σ1.5	0.9169 (0.2718)	0.042 (0.0917)	0.892 (0.2745)	0.2116 (0.202)
N-St2-σ1.5	0.7862 (0.3356)	0.153 (0.1292)	0.7377 (0.3234)	0.328 (0.2165)
N-All-σ1.5	0.8899 (0.2706)	0.0977 (0.0907)	0.8343 (0.2713)	0.3228 (0.208)
N-Str-σ1.5	0.8912 (0.2693)	0.1053 (0.0937)	0.8503 (0.2722)	0.275 (0.1863)
P-St1-σ0.5	0.9989 (0.0019)	0.067 (0.1117)	0.983 (0.0235)	0.4988 (0.2343)
P-St2-σ0.5	0.9344 (0.0718)	0.288 (0.1565)	0.8891 (0.0903)	0.5316 (0.2037)
P-All-σ0.5	0.9895 (0.0149)	0.1754 (0.1078)	0.9565 (0.0515)	0.5998 (0.1669)
P-Str-σ0.5	0.988 (0.0164)	0.1924 (0.112)	0.967 (0.0341)	0.5141 (0.1843)
P-St1-σ1.0	0.9373 (0.2381)	0.0482 (0.0944)	0.9166 (0.2346)	0.2949 (0.2283)
P-St2-σ1.0	0.8685 (0.2233)	0.2045 (0.1497)	0.8232 (0.2228)	0.3992 (0.2207)
P-All-σ1.0	0.9388 (0.1861)	0.1264 (0.1028)	0.8884 (0.1929)	0.412 (0.2064)
P-Str-σ1.0	0.9391 (0.1806)	0.1363 (0.1041)	0.9073 (0.1821)	0.3526 (0.1939)
P-St1-σ1.5	0.8869 (0.3134)	0.0408 (0.0896)	0.8617 (0.313)	0.212 (0.2032)
P-St2-σ1.5	0.774 (0.3418)	0.1637 (0.135)	0.7258 (0.3259)	0.3395 (0.2219)
P-All-σ1.5	0.8576 (0.3089)	0.1018 (0.0918)	0.8061 (0.3046)	0.3199 (0.2061)
P-Str-σ1.5	0.8764 (0.2839)	0.1111 (0.0961)	0.8345 (0.284)	0.2814 (0.1871)
S-St1-σ0.5	0.9979 (0.0068)	0.0537 (0.1832)	0.9901 (0.015)	0.1843 (0.2894)
S-St2-σ0.5	0.9365 (0.0285)	0.3892 (0.1445)	0.897 (0.0354)	0.6507 (0.1257)
S-All-σ0.5	0.9538 (0.0208)	0.3554 (0.1409)	0.917 (0.028)	0.6537 (0.1266)
S-Str-σ0.5	0.9538 (0.0207)	0.3573 (0.1401)	0.9225 (0.0271)	0.6088 (0.1262)
S-St1-σ1.0	0.8836 (0.3149)	0.0181 (0.0835)	0.8733 (0.3129)	0.0706 (0.17)
S-St2-σ1.0	0.8607 (0.0946)	0.256 (0.146)	0.7932 (0.1023)	0.4028 (0.1539)
S-All-σ1.0	0.8813 (0.0892)	0.2325 (0.135)	0.8069 (0.0998)	0.4077 (0.1513)
S-Str-σ1.0	0.887 (0.0873)	0.2347 (0.1354)	0.8309 (0.0889)	0.3736 (0.1449)
S-St1-σ1.5	0.677 (0.4671)	0.014 (0.0804)	0.6696 (0.4633)	0.0415 (0.1543)
S-St2-σ1.5	0.7794 (0.2346)	0.1723 (0.1438)	0.7191 (0.2274)	0.2673 (0.1913)
S-All-σ1.5	0.7873 (0.2457)	0.1503 (0.1298)	0.7233 (0.2344)	0.2485 (0.1814)
S-Str-σ1.5	0.8177 (0.1986)	0.1587 (0.1315)	0.7632 (0.1963)	0.2464 (0.1745)

Table S2.C. The estimated FDR is 0.5 using the method: BH

	H1		H1-Corr	
	FDR	sensitivity	FDR	sensitivity
N-St1-σ0.5	0.9994 (7e-04)	0.1113 (0.1375)	0.9919 (0.01)	0.681 (0.2013)
N-St2-σ0.5	0.9668 (0.0508)	0.317 (0.1595)	0.933 (0.0684)	0.6412 (0.1889)
N-All-σ0.5	0.9965 (0.0031)	0.213 (0.1322)	0.9817 (0.0247)	0.7443 (0.1455)
N-Str-σ0.5	0.9961 (0.0033)	0.2291 (0.1295)	0.9853 (0.0149)	0.6571 (0.1663)
N-St1-σ1.0	0.9486 (0.2187)	0.0566 (0.1109)	0.9323 (0.2159)	0.4112 (0.253)
N-St2-σ1.0	0.9042 (0.207)	0.2159 (0.1591)	0.85 (0.2131)	0.4835 (0.24)
N-All-σ1.0	0.9397 (0.2171)	0.1325 (0.1084)	0.904 (0.2148)	0.5053 (0.2262)
N-Str-σ1.0	0.9568 (0.1707)	0.1475 (0.1153)	0.9288 (0.1726)	0.4525 (0.2201)
N-St1-σ1.5	0.9279 (0.2559)	0.0479 (0.0965)	0.9068 (0.2593)	0.2816 (0.2357)
N-St2-σ1.5	0.851 (0.2941)	0.1687 (0.1364)	0.8029 (0.2831)	0.4119 (0.2332)
N-All-σ1.5	0.9114 (0.2551)	0.1062 (0.0964)	0.8606 (0.2585)	0.3928 (0.235)
N-Str-σ1.5	0.925 (0.236)	0.1175 (0.1016)	0.8937 (0.233)	0.3527 (0.2125)
P-St1-σ0.5	0.9994 (9e-04)	0.0993 (0.1333)	0.9902 (0.0121)	0.6503 (0.2184)
P-St2-σ0.5	0.9606 (0.0447)	0.3566 (0.1741)	0.9261 (0.0689)	0.643 (0.1883)
P-All-σ0.5	0.9952 (0.0044)	0.2224 (0.1327)	0.9772 (0.0323)	0.7266 (0.1497)
P-Str-σ0.5	0.9945 (0.0046)	0.2454 (0.1325)	0.9815 (0.0201)	0.6446 (0.1681)
P-St1-σ1.0	0.9685 (0.1712)	0.054 (0.1083)	0.9457 (0.1805)	0.3877 (0.2458)
P-St2-σ1.0	0.907 (0.1866)	0.2345 (0.1609)	0.8539 (0.1955)	0.4888 (0.2317)
P-All-σ1.0	0.9705 (0.1133)	0.1387 (0.1108)	0.9317 (0.1275)	0.4981 (0.2233)
P-Str-σ1.0	0.9738 (0.1015)	0.1561 (0.1131)	0.9412 (0.121)	0.4445 (0.2126)
P-St1-σ1.5	0.9559 (0.1972)	0.0464 (0.0935)	0.9342 (0.2043)	0.2775 (0.2334)
P-St2-σ1.5	0.8569 (0.28)	0.1831 (0.1426)	0.8063 (0.2709)	0.4204 (0.2276)
P-All-σ1.5	0.9427 (0.1963)	0.1115 (0.0981)	0.8831 (0.2142)	0.3948 (0.2294)
P-Str-σ1.5	0.941 (0.1956)	0.1246 (0.1012)	0.9066 (0.196)	0.3561 (0.2052)
S-St1-σ0.5	0.9977 (0.0065)	0.0701 (0.1991)	0.992 (0.0118)	0.2183 (0.3142)
S-St2-σ0.5	0.9507 (0.0202)	0.4287 (0.1358)	0.9201 (0.0275)	0.7058 (0.1104)
S-All-σ0.5	0.9644 (0.0137)	0.3916 (0.1303)	0.936 (0.0209)	0.7081 (0.1057)
S-Str-σ0.5	0.9643 (0.014)	0.3946 (0.1309)	0.9402 (0.0202)	0.6605 (0.1101)
S-St1-σ1.0	0.976 (0.1415)	0.0206 (0.0866)	0.9677 (0.142)	0.1024 (0.2175)
S-St2-σ1.0	0.9154 (0.0483)	0.2984 (0.1475)	0.8599 (0.0549)	0.5215 (0.1599)
S-All-σ1.0	0.936 (0.0374)	0.2696 (0.1403)	0.8796 (0.0482)	0.5289 (0.1491)
S-Str-σ1.0	0.9353 (0.0387)	0.2731 (0.1371)	0.89 (0.0459)	0.4836 (0.1503)
S-St1-σ1.5	0.8485 (0.3583)	0.014 (0.0804)	0.8407 (0.3562)	0.0616 (0.1882)
S-St2-σ1.5	0.8761 (0.1424)	0.1984 (0.1542)	0.8066 (0.1461)	0.3615 (0.2098)
S-All-σ1.5	0.8806 (0.1796)	0.1768 (0.1393)	0.8072 (0.1754)	0.3572 (0.2073)
S-Str-σ1.5	0.9011 (0.1335)	0.1824 (0.1409)	0.842 (0.1357)	0.3346 (0.1943)

Table S2.D. The estimated FDR is 0.05 using the method: BUM

	H1		H1-Corr	
	FDR	sensitivity	FDR	sensitivity
N-St1-σ0.5	0.9882 (0.0999)	0.0586 (0.112)	0.9589 (0.1031)	0.4997 (0.2471)
N-St2-σ0.5	0.9114 (0.1064)	0.22 (0.1471)	0.8477 (0.1108)	0.472 (0.2117)
N-All-σ0.5	0.9824 (0.0299)	0.1442 (0.1029)	0.9245 (0.0882)	0.5795 (0.1867)
N-Str-σ0.5	0.9799 (0.0416)	0.1496 (0.1037)	0.9442 (0.0559)	0.4811 (0.1986)
N-St1-σ1.0	0.8861 (0.3132)	0.0419 (0.0832)	0.8557 (0.3053)	0.2986 (0.238)
N-St2-σ1.0	0.7882 (0.3159)	0.169 (0.138)	0.7358 (0.2963)	0.3698 (0.2159)
N-All-σ1.0	0.8654 (0.3074)	0.1104 (0.0948)	0.8079 (0.2945)	0.4002 (0.2168)
N-Str-σ1.0	0.8645 (0.3071)	0.1139 (0.0941)	0.8276 (0.2965)	0.3393 (0.2026)
N-St1-σ1.5	0.7458 (0.4329)	0.0364 (0.0844)	0.7251 (0.4213)	0.2052 (0.226)
N-St2-σ1.5	0.7187 (0.373)	0.1374 (0.1213)	0.6633 (0.3455)	0.3072 (0.2148)
N-All-σ1.5	0.7459 (0.4031)	0.0882 (0.0867)	0.7015 (0.3817)	0.2948 (0.2242)
N-Str-σ1.5	0.7794 (0.3785)	0.0938 (0.0875)	0.7382 (0.3603)	0.2625 (0.1976)
P-St1-σ0.5	0.9779 (0.1405)	0.0571 (0.1066)	0.9451 (0.1468)	0.4724 (0.2438)
P-St2-σ0.5	0.8987 (0.1189)	0.2391 (0.145)	0.8332 (0.1263)	0.487 (0.2118)
P-All-σ0.5	0.9696 (0.1065)	0.1518 (0.1008)	0.9108 (0.1301)	0.5655 (0.1852)
P-Str-σ0.5	0.9704 (0.0734)	0.1593 (0.0999)	0.9319 (0.087)	0.4783 (0.1988)
P-St1-σ1.0	0.8861 (0.3132)	0.0409 (0.0834)	0.8542 (0.3057)	0.2898 (0.2313)
P-St2-σ1.0	0.7928 (0.3066)	0.1804 (0.1487)	0.7396 (0.2879)	0.38 (0.2224)
P-All-σ1.0	0.8628 (0.307)	0.1143 (0.0955)	0.8045 (0.2941)	0.3961 (0.2137)
P-Str-σ1.0	0.872 (0.2944)	0.1198 (0.0996)	0.8339 (0.2848)	0.3406 (0.2045)
P-St1-σ1.5	0.7457 (0.4328)	0.0364 (0.0844)	0.7219 (0.4201)	0.204 (0.2238)
P-St2-σ1.5	0.7157 (0.3697)	0.1463 (0.1253)	0.6604 (0.3422)	0.3136 (0.2174)
P-All-σ1.5	0.7398 (0.4019)	0.0933 (0.0873)	0.6972 (0.3795)	0.2923 (0.2209)
P-Str-σ1.5	0.7797 (0.3723)	0.0986 (0.0888)	0.7382 (0.3539)	0.2655 (0.1973)
S-St1-σ0.5	0.9977 (0.0092)	0.0312 (0.121)	0.9774 (0.0381)	0.2039 (0.3125)
S-St2-σ0.5	0.9006 (0.0488)	0.3446 (0.1462)	0.832 (0.0537)	0.6332 (0.1352)
S-All-σ0.5	0.9271 (0.0371)	0.3128 (0.1397)	0.8582 (0.0456)	0.6575 (0.1211)
S-Str-σ0.5	0.9258 (0.0375)	0.3159 (0.1407)	0.8676 (0.0451)	0.6006 (0.1263)
S-St1-σ1.0	0.6664 (0.4706)	0.0125 (0.0743)	0.6544 (0.4655)	0.0458 (0.1601)
S-St2-σ1.0	0.7767 (0.1496)	0.2165 (0.1337)	0.679 (0.1414)	0.361 (0.1562)
S-All-σ1.0	0.7972 (0.1572)	0.1946 (0.1202)	0.689 (0.1467)	0.3659 (0.1608)
S-Str-σ1.0	0.7981 (0.1487)	0.1975 (0.1234)	0.7078 (0.1437)	0.332 (0.1484)
S-St1-σ1.5	0.368 (0.483)	0.005 (0.05)	0.3591 (0.4734)	0.0275 (0.1346)
S-St2-σ1.5	0.635 (0.3385)	0.1247 (0.1107)	0.5525 (0.2989)	0.2126 (0.1466)
S-All-σ1.5	0.6572 (0.3435)	0.1111 (0.0999)	0.5585 (0.3016)	0.2126 (0.1534)
S-Str-σ1.5	0.6541 (0.3372)	0.1142 (0.1017)	0.5754 (0.3026)	0.1951 (0.1371)

Table S2.E. The estimated FDR is 0.25 using the method: BUM

	H1		H1-Corr	
	FDR	sensitivity	FDR	sensitivity
N-St1-σ0.5	0.9989 (0.0019)	0.0696 (0.1167)	0.9789 (0.0276)	0.65 (0.2207)
N-St2-σ0.5	0.9402 (0.0673)	0.2561 (0.1495)	0.8786 (0.0908)	0.5737 (0.2104)
N-All-σ0.5	0.9917 (0.0085)	0.162 (0.1099)	0.953 (0.0553)	0.7081 (0.1542)
N-Str-σ0.5	0.9911 (0.0085)	0.1757 (0.1106)	0.9646 (0.0346)	0.6047 (0.1819)
N-St1-σ1.0	0.9074 (0.2869)	0.0492 (0.0968)	0.8801 (0.2801)	0.3848 (0.2652)
N-St2-σ1.0	0.834 (0.2836)	0.1859 (0.1412)	0.7807 (0.2706)	0.4398 (0.2469)
N-All-σ1.0	0.8829 (0.2967)	0.1189 (0.0994)	0.8303 (0.2864)	0.4797 (0.2379)
N-Str-σ1.0	0.9067 (0.2557)	0.1267 (0.1008)	0.8714 (0.2511)	0.4154 (0.2322)
N-St1-σ1.5	0.7769 (0.4147)	0.0408 (0.0896)	0.751 (0.403)	0.2788 (0.2471)
N-St2-σ1.5	0.7717 (0.3493)	0.1518 (0.1286)	0.7152 (0.3289)	0.377 (0.2446)
N-All-σ1.5	0.7903 (0.3769)	0.0964 (0.0908)	0.738 (0.3584)	0.3693 (0.2474)
N-Str-σ1.5	0.8255 (0.3507)	0.1044 (0.0933)	0.7827 (0.3376)	0.3331 (0.2246)
P-St1-σ0.5	0.9888 (0.0999)	0.0635 (0.1109)	0.9694 (0.0996)	0.6153 (0.2358)
P-St2-σ0.5	0.9307 (0.0649)	0.2824 (0.156)	0.8631 (0.112)	0.5799 (0.197)
P-All-σ0.5	0.9895 (0.0115)	0.1693 (0.1061)	0.9462 (0.059)	0.6922 (0.1603)
P-Str-σ0.5	0.9864 (0.0233)	0.1877 (0.1116)	0.9528 (0.0754)	0.5935 (0.1815)
P-St1-σ1.0	0.9073 (0.2868)	0.0482 (0.0944)	0.8787 (0.2803)	0.3695 (0.2619)
P-St2-σ1.0	0.8349 (0.2728)	0.2019 (0.1502)	0.7797 (0.2609)	0.4483 (0.2448)
P-All-σ1.0	0.8809 (0.2962)	0.1252 (0.1041)	0.8268 (0.2863)	0.4754 (0.2364)
P-Str-σ1.0	0.9119 (0.2378)	0.1349 (0.1046)	0.8739 (0.2355)	0.4133 (0.2276)
P-St1-σ1.5	0.7668 (0.4212)	0.0414 (0.0895)	0.7409 (0.4088)	0.2751 (0.2482)
P-St2-σ1.5	0.7554 (0.3579)	0.1644 (0.1365)	0.7022 (0.3368)	0.3819 (0.2502)
P-All-σ1.5	0.7801 (0.3839)	0.1006 (0.0932)	0.7293 (0.3632)	0.3704 (0.2466)
P-Str-σ1.5	0.8159 (0.3537)	0.1116 (0.0968)	0.7745 (0.3402)	0.3332 (0.2265)
S-St1-σ0.5	0.9978 (0.0074)	0.0537 (0.1832)	0.9836 (0.0226)	0.2709 (0.3515)
S-St2-σ0.5	0.9333 (0.03)	0.3808 (0.1433)	0.8811 (0.0378)	0.7114 (0.1206)
S-All-σ0.5	0.9517 (0.0217)	0.3492 (0.1407)	0.9024 (0.0299)	0.7312 (0.1034)
S-Str-σ0.5	0.9512 (0.0221)	0.3498 (0.1401)	0.9083 (0.0294)	0.6785 (0.118)
S-St1-σ1.0	0.7776 (0.4153)	0.0125 (0.0743)	0.7614 (0.4089)	0.0981 (0.2193)
S-St2-σ1.0	0.8453 (0.0999)	0.2543 (0.1444)	0.7521 (0.1032)	0.4562 (0.161)
S-All-σ1.0	0.8716 (0.0916)	0.2286 (0.1321)	0.7687 (0.1002)	0.4705 (0.1569)
S-Str-σ1.0	0.8691 (0.0941)	0.2325 (0.1349)	0.7852 (0.1022)	0.4254 (0.1569)
S-St1-σ1.5	0.5074 (0.5002)	0.01 (0.0704)	0.4985 (0.4928)	0.0442 (0.1626)
S-St2-σ1.5	0.7419 (0.2763)	0.1669 (0.1462)	0.6497 (0.2489)	0.3004 (0.2003)
S-All-σ1.5	0.77 (0.2659)	0.1456 (0.124)	0.6639 (0.2395)	0.2934 (0.1902)
S-Str-σ1.5	0.7796 (0.253)	0.153 (0.1341)	0.6932 (0.2349)	0.2764 (0.1862)

Table S2.F. The estimated FDR is 0.5 using the method: BUM

	H1		H1-Corr	
	FDR	sensitivity	FDR	sensitivity
N-St1-σ0.5	0.9992 (0.0011)	0.0896 (0.123)	0.9868 (0.0165)	0.7753 (0.1773)
N-St2-σ0.5	0.9589 (0.0566)	0.2923 (0.1522)	0.9107 (0.0763)	0.6636 (0.1879)
N-All-σ0.5	0.9953 (0.004)	0.1889 (0.1193)	0.9731 (0.0301)	0.8033 (0.1243)
N-Str-σ0.5	0.9948 (0.0042)	0.2057 (0.1177)	0.9779 (0.0209)	0.7101 (0.1446)
N-St1-σ1.0	0.9184 (0.2722)	0.0526 (0.1004)	0.8969 (0.2669)	0.4852 (0.2769)
N-St2-σ1.0	0.8636 (0.2688)	0.2123 (0.156)	0.8101 (0.261)	0.5277 (0.2719)
N-All-σ1.0	0.9094 (0.2698)	0.1278 (0.1049)	0.8629 (0.2636)	0.5716 (0.2466)
N-Str-σ1.0	0.9218 (0.2398)	0.1434 (0.1108)	0.8897 (0.2375)	0.5104 (0.2533)
N-St1-σ1.5	0.7979 (0.401)	0.0478 (0.0987)	0.7764 (0.391)	0.3738 (0.2894)
N-St2-σ1.5	0.839 (0.3073)	0.1709 (0.14)	0.7767 (0.2942)	0.4672 (0.2642)
N-All-σ1.5	0.8133 (0.3718)	0.1058 (0.0964)	0.7625 (0.3597)	0.4591 (0.2854)
N-Str-σ1.5	0.8798 (0.2994)	0.1184 (0.1034)	0.8318 (0.2938)	0.4237 (0.2523)
P-St1-σ0.5	0.9891 (0.0999)	0.0814 (0.1213)	0.9757 (0.0994)	0.741 (0.1992)
P-St2-σ0.5	0.9508 (0.052)	0.3293 (0.1624)	0.8999 (0.0919)	0.6648 (0.1809)
P-All-σ0.5	0.9937 (0.0055)	0.1975 (0.1177)	0.9667 (0.0396)	0.7905 (0.1189)
P-Str-σ0.5	0.9905 (0.0283)	0.2219 (0.1205)	0.9689 (0.0648)	0.6962 (0.1479)
P-St1-σ1.0	0.9183 (0.2722)	0.0521 (0.1053)	0.8945 (0.267)	0.4694 (0.2774)
P-St2-σ1.0	0.8831 (0.2361)	0.2319 (0.1623)	0.8194 (0.2355)	0.543 (0.2646)
P-All-σ1.0	0.918 (0.2536)	0.1329 (0.1096)	0.87 (0.2491)	0.5584 (0.2475)
P-Str-σ1.0	0.9424 (0.1954)	0.1536 (0.1126)	0.9023 (0.1999)	0.5111 (0.247)
P-St1-σ1.5	0.7879 (0.4083)	0.0455 (0.0963)	0.7646 (0.3974)	0.3721 (0.2897)
P-St2-σ1.5	0.8358 (0.3048)	0.185 (0.1434)	0.7787 (0.2921)	0.4655 (0.2631)
P-All-σ1.5	0.8028 (0.3795)	0.1097 (0.0984)	0.7602 (0.3634)	0.4528 (0.2835)
P-Str-σ1.5	0.8833 (0.2863)	0.1248 (0.1023)	0.8385 (0.2812)	0.4213 (0.2555)
S-St1-σ0.5	0.9971 (0.0074)	0.0776 (0.2042)	0.9867 (0.0181)	0.3251 (0.3793)
S-St2-σ0.5	0.9488 (0.0211)	0.4248 (0.1348)	0.9104 (0.0282)	0.7673 (0.1067)
S-All-σ0.5	0.9632 (0.0144)	0.3896 (0.1311)	0.927 (0.0221)	0.7875 (0.0878)
S-Str-σ0.5	0.9628 (0.0148)	0.393 (0.1305)	0.9316 (0.0214)	0.7339 (0.1026)
S-St1-σ1.0	0.8683 (0.3375)	0.0158 (0.0809)	0.8553 (0.3339)	0.1321 (0.2596)
S-St2-σ1.0	0.9012 (0.0572)	0.2841 (0.1477)	0.8213 (0.0677)	0.5596 (0.1594)
S-All-σ1.0	0.9209 (0.0497)	0.2533 (0.1381)	0.8372 (0.0647)	0.5715 (0.1535)
S-Str-σ1.0	0.9208 (0.051)	0.2591 (0.1383)	0.8522 (0.0608)	0.5223 (0.1521)
S-St1-σ1.5	0.5883 (0.493)	0.014 (0.0804)	0.5786 (0.4859)	0.061 (0.1759)
S-St2-σ1.5	0.8266 (0.2211)	0.1899 (0.156)	0.729 (0.2086)	0.3844 (0.2146)
S-All-σ1.5	0.8405 (0.2315)	0.1667 (0.1413)	0.7401 (0.2191)	0.3865 (0.2271)
S-Str-σ1.5	0.8607 (0.1863)	0.1746 (0.1425)	0.7701 (0.1847)	0.3547 (0.1992)

Table S2.G. The estimated FDR is 0.05 using the method: fdrtool

	H1		H1-Corr	
	FDR	sensitivity	FDR	sensitivity
N-St1-σ0.5	0.9982 (0.0035)	0.0586 (0.112)	0.9766 (0.0292)	0.3679 (0.2287)
N-St2-σ0.5	0.913 (0.1059)	0.2212 (0.1469)	0.8625 (0.1126)	0.4232 (0.1958)
N-All-σ0.5	0.9815 (0.0302)	0.1449 (0.1022)	0.9356 (0.0786)	0.4843 (0.1823)
N-Str-σ0.5	0.98 (0.036)	0.1503 (0.1035)	0.9526 (0.0492)	0.3952 (0.1755)
N-St1-σ1.0	0.8961 (0.3003)	0.0419 (0.0832)	0.8742 (0.2962)	0.2238 (0.2063)
N-St2-σ1.0	0.7963 (0.3129)	0.1686 (0.1397)	0.7577 (0.2996)	0.3264 (0.2005)
N-All-σ1.0	0.8754 (0.2949)	0.1098 (0.0938)	0.8302 (0.2841)	0.3414 (0.1939)
N-Str-σ1.0	0.876 (0.2949)	0.1136 (0.0954)	0.8487 (0.287)	0.2817 (0.1776)
N-St1-σ1.5	0.7859 (0.4074)	0.0359 (0.0851)	0.7725 (0.4008)	0.1482 (0.1845)
N-St2-σ1.5	0.7346 (0.3662)	0.1364 (0.1208)	0.6863 (0.3481)	0.2679 (0.1944)
N-All-σ1.5	0.7733 (0.3837)	0.0884 (0.0873)	0.7284 (0.367)	0.2531 (0.1939)
N-Str-σ1.5	0.8061 (0.3528)	0.093 (0.0879)	0.7725 (0.3437)	0.2154 (0.1656)
P-St1-σ0.5	0.9879 (0.0999)	0.0567 (0.1058)	0.9655 (0.102)	0.3571 (0.2244)
P-St2-σ0.5	0.8998 (0.1195)	0.2395 (0.1453)	0.8513 (0.1232)	0.4294 (0.1945)
P-All-σ0.5	0.9746 (0.0603)	0.1516 (0.1011)	0.9269 (0.0912)	0.478 (0.1811)
P-Str-σ0.5	0.9715 (0.0745)	0.1593 (0.0997)	0.9433 (0.0781)	0.3945 (0.1737)
P-St1-σ1.0	0.896 (0.3003)	0.0413 (0.0833)	0.8771 (0.296)	0.2112 (0.1954)
P-St2-σ1.0	0.8083 (0.2869)	0.1819 (0.1477)	0.7708 (0.2756)	0.3365 (0.2015)
P-All-σ1.0	0.8726 (0.2946)	0.1153 (0.0966)	0.8302 (0.2834)	0.3339 (0.1902)
P-Str-σ1.0	0.8968 (0.2543)	0.1211 (0.0989)	0.8715 (0.2481)	0.282 (0.1758)
P-St1-σ1.5	0.7661 (0.4209)	0.0351 (0.084)	0.7536 (0.4143)	0.1446 (0.1826)
P-St2-σ1.5	0.7305 (0.3637)	0.1454 (0.1251)	0.6841 (0.3462)	0.2723 (0.193)
P-All-σ1.5	0.7611 (0.3902)	0.0923 (0.0883)	0.7168 (0.3733)	0.2511 (0.1912)
P-Str-σ1.5	0.7994 (0.3549)	0.0975 (0.0885)	0.7675 (0.3456)	0.2161 (0.1629)
S-St1-σ0.5	0.9974 (0.0102)	0.0312 (0.121)	0.9846 (0.03)	0.1298 (0.2456)
S-St2-σ0.5	0.8908 (0.0523)	0.3372 (0.1448)	0.8319 (0.0564)	0.5573 (0.1443)
S-All-σ0.5	0.9196 (0.0395)	0.3088 (0.1388)	0.8619 (0.0454)	0.5648 (0.1348)
S-Str-σ0.5	0.9198 (0.0395)	0.3088 (0.1388)	0.8718 (0.0458)	0.5195 (0.1397)
S-St1-σ1.0	0.6664 (0.4706)	0.0125 (0.0743)	0.6601 (0.4676)	0.0312 (0.1321)
S-St2-σ1.0	0.7781 (0.1504)	0.2163 (0.1325)	0.707 (0.1554)	0.314 (0.1478)
S-All-σ1.0	0.7987 (0.1583)	0.1933 (0.1177)	0.7213 (0.1618)	0.3068 (0.1513)
S-Str-σ1.0	0.8015 (0.1496)	0.1972 (0.1222)	0.736 (0.1552)	0.2862 (0.1361)
S-St1-σ1.5	0.3886 (0.4886)	0.005 (0.05)	0.3846 (0.4845)	0.0183 (0.1158)
S-St2-σ1.5	0.6582 (0.3301)	0.1221 (0.1101)	0.6092 (0.3175)	0.1719 (0.1388)
S-All-σ1.5	0.6725 (0.3325)	0.1104 (0.1005)	0.6063 (0.3144)	0.1726 (0.1352)
S-Str-σ1.5	0.6783 (0.3274)	0.1118 (0.1011)	0.631 (0.317)	0.1565 (0.1263)

Table S2.H. The estimated FDR is 0.25 using the method: fdrtool

	H1		H1-Corr	
	FDR	sensitivity	FDR	sensitivity
N-St1-σ0.5	0.9991 (0.0015)	0.0744 (0.118)	0.9855 (0.0183)	0.5421 (0.2385)
N-St2-σ0.5	0.9492 (0.0593)	0.2673 (0.1537)	0.9039 (0.0774)	0.5437 (0.2033)
N-All-σ0.5	0.9923 (0.01)	0.1711 (0.117)	0.9627 (0.0489)	0.6215 (0.1702)
N-Str-σ0.5	0.9917 (0.0098)	0.1845 (0.115)	0.9728 (0.0247)	0.5402 (0.1864)
N-St1-σ1.0	0.9276 (0.2558)	0.0499 (0.098)	0.9089 (0.2519)	0.306 (0.2393)
N-St2-σ1.0	0.8351 (0.2947)	0.1874 (0.1448)	0.7929 (0.2849)	0.3909 (0.2263)
N-All-σ1.0	0.9138 (0.2529)	0.1198 (0.1006)	0.8703 (0.2476)	0.4183 (0.2133)
N-Str-σ1.0	0.923 (0.2355)	0.1276 (0.1031)	0.8956 (0.2309)	0.3521 (0.2037)
N-St1-σ1.5	0.8471 (0.3577)	0.0408 (0.0914)	0.8293 (0.3517)	0.211 (0.2044)
N-St2-σ1.5	0.7821 (0.3447)	0.1517 (0.1301)	0.7319 (0.3314)	0.3336 (0.2224)
N-All-σ1.5	0.8425 (0.3321)	0.0974 (0.0914)	0.798 (0.3192)	0.3205 (0.2126)
N-Str-σ1.5	0.8633 (0.309)	0.1039 (0.0944)	0.8262 (0.3064)	0.2778 (0.1919)
P-St1-σ0.5	0.9989 (0.0018)	0.0686 (0.1136)	0.9837 (0.0225)	0.5068 (0.2384)
P-St2-σ0.5	0.9379 (0.0683)	0.2952 (0.1595)	0.8934 (0.0855)	0.5431 (0.2018)
P-All-σ0.5	0.9899 (0.0141)	0.1794 (0.1103)	0.9582 (0.0498)	0.6062 (0.1695)
P-Str-σ0.5	0.9885 (0.0156)	0.1972 (0.1141)	0.9678 (0.0319)	0.5242 (0.1839)
P-St1-σ1.0	0.9274 (0.2558)	0.0482 (0.0944)	0.9061 (0.2517)	0.3023 (0.2344)
P-St2-σ1.0	0.8599 (0.2448)	0.2043 (0.1523)	0.8142 (0.2428)	0.4043 (0.2269)
P-All-σ1.0	0.9259 (0.2203)	0.1267 (0.1033)	0.8747 (0.2242)	0.4181 (0.2083)
P-Str-σ1.0	0.9358 (0.197)	0.1362 (0.1054)	0.9037 (0.1976)	0.3587 (0.2001)
P-St1-σ1.5	0.8171 (0.3848)	0.0402 (0.0892)	0.7987 (0.3776)	0.2105 (0.2047)
P-St2-σ1.5	0.7728 (0.348)	0.164 (0.1367)	0.7254 (0.3322)	0.3375 (0.2215)
P-All-σ1.5	0.803 (0.3685)	0.1011 (0.0929)	0.7621 (0.3536)	0.319 (0.2119)
P-Str-σ1.5	0.8375 (0.3315)	0.1109 (0.0969)	0.8018 (0.3241)	0.2796 (0.1885)
S-St1-σ0.5	0.9974 (0.0084)	0.0537 (0.1832)	0.9891 (0.0171)	0.1718 (0.2864)
S-St2-σ0.5	0.9237 (0.0358)	0.3677 (0.1456)	0.8773 (0.0414)	0.6187 (0.1312)
S-All-σ0.5	0.9448 (0.0255)	0.338 (0.1417)	0.9014 (0.0308)	0.6264 (0.1246)
S-Str-σ0.5	0.945 (0.0256)	0.338 (0.1417)	0.9082 (0.0312)	0.5787 (0.1307)
S-St1-σ1.0	0.8374 (0.3675)	0.0158 (0.0809)	0.827 (0.3644)	0.0684 (0.1694)
S-St2-σ1.0	0.8578 (0.0844)	0.2596 (0.1421)	0.7929 (0.0914)	0.4064 (0.1482)
S-All-σ1.0	0.8849 (0.0775)	0.2323 (0.1345)	0.8106 (0.0839)	0.4115 (0.1434)
S-Str-σ1.0	0.8849 (0.0777)	0.2371 (0.1327)	0.8291 (0.0818)	0.3761 (0.1406)
S-St1-σ1.5	0.5978 (0.4908)	0.01 (0.0704)	0.5906 (0.486)	0.0375 (0.15)
S-St2-σ1.5	0.7676 (0.2549)	0.1719 (0.1476)	0.7094 (0.2458)	0.2662 (0.1982)
S-All-σ1.5	0.7862 (0.2527)	0.1488 (0.1315)	0.7177 (0.2429)	0.2519 (0.1818)
S-Str-σ1.5	0.8094 (0.2181)	0.1577 (0.1352)	0.7559 (0.2143)	0.2451 (0.1809)

Table S2.I. The estimated FDR is 0.5 using the method: fdrtool

	H1		H1-Corr	
	FDR	sensitivity	FDR	sensitivity
N-St1-σ0.5	0.9994 (7e-04)	0.122 (0.1398)	0.9925 (0.0096)	0.7032 (0.2014)
N-St2-σ0.5	0.9722 (0.0356)	0.3338 (0.1716)	0.9404 (0.0598)	0.6679 (0.1844)
N-All-σ0.5	0.9968 (0.0028)	0.224 (0.1363)	0.9829 (0.0237)	0.7655 (0.1394)
N-Str-σ0.5	0.9964 (0.0031)	0.2431 (0.1372)	0.9864 (0.0145)	0.6829 (0.1631)
N-St1-σ1.0	0.9486 (0.2187)	0.0597 (0.1178)	0.9337 (0.2161)	0.4217 (0.2615)
N-St2-σ1.0	0.8883 (0.2533)	0.2251 (0.1657)	0.8429 (0.2521)	0.493 (0.2561)
N-All-σ1.0	0.9393 (0.2175)	0.135 (0.1124)	0.9032 (0.2175)	0.5198 (0.2325)
N-Str-σ1.0	0.9498 (0.1953)	0.1538 (0.1193)	0.927 (0.1934)	0.462 (0.2319)
N-St1-σ1.5	0.8466 (0.3578)	0.0496 (0.1015)	0.8319 (0.3521)	0.2928 (0.2479)
N-St2-σ1.5	0.8475 (0.3062)	0.1733 (0.1433)	0.8003 (0.2933)	0.4243 (0.24)
N-All-σ1.5	0.8552 (0.3337)	0.108 (0.0977)	0.8179 (0.3237)	0.4009 (0.246)
N-Str-σ1.5	0.8959 (0.2839)	0.1205 (0.1063)	0.8627 (0.2778)	0.3644 (0.2189)
P-St1-σ0.5	0.9994 (8e-04)	0.1048 (0.1374)	0.9911 (0.0112)	0.6687 (0.2112)
P-St2-σ0.5	0.9647 (0.0394)	0.376 (0.1802)	0.9321 (0.0704)	0.6739 (0.1866)
P-All-σ0.5	0.9956 (0.004)	0.2326 (0.1367)	0.9791 (0.0307)	0.7416 (0.1498)
P-Str-σ0.5	0.995 (0.004)	0.2587 (0.1373)	0.9833 (0.018)	0.6693 (0.1666)
P-St1-σ1.0	0.9586 (0.1967)	0.0543 (0.1091)	0.9366 (0.2038)	0.4014 (0.2484)
P-St2-σ1.0	0.8969 (0.222)	0.2419 (0.1634)	0.8458 (0.2287)	0.5036 (0.2455)
P-All-σ1.0	0.953 (0.1765)	0.1394 (0.1113)	0.9166 (0.1781)	0.5097 (0.2267)
P-Str-σ1.0	0.9642 (0.1418)	0.1608 (0.1147)	0.9338 (0.1551)	0.4597 (0.2203)
P-St1-σ1.5	0.8564 (0.3477)	0.0473 (0.0981)	0.8414 (0.3421)	0.2831 (0.2407)
P-St2-σ1.5	0.8536 (0.2928)	0.186 (0.1463)	0.8068 (0.2817)	0.4313 (0.233)
P-All-σ1.5	0.865 (0.3223)	0.1116 (0.1)	0.829 (0.3132)	0.3928 (0.2447)
P-Str-σ1.5	0.9119 (0.2536)	0.1265 (0.1034)	0.8783 (0.2493)	0.3643 (0.2104)
S-St1-σ0.5	0.9974 (0.0076)	0.0653 (0.1979)	0.9907 (0.0134)	0.2008 (0.3017)
S-St2-σ0.5	0.9391 (0.0257)	0.3961 (0.1416)	0.9012 (0.0309)	0.6645 (0.1184)
S-All-σ0.5	0.9561 (0.0181)	0.3648 (0.1372)	0.9215 (0.0232)	0.67 (0.1214)
S-Str-σ0.5	0.9563 (0.0182)	0.3648 (0.1372)	0.9267 (0.0229)	0.6217 (0.1192)
S-St1-σ1.0	0.9183 (0.2723)	0.0158 (0.0809)	0.9102 (0.2707)	0.0977 (0.2171)
S-St2-σ1.0	0.9133 (0.0482)	0.2974 (0.145)	0.8575 (0.0556)	0.5149 (0.1573)
S-All-σ1.0	0.9341 (0.0362)	0.2659 (0.1403)	0.8732 (0.0467)	0.5183 (0.1411)
S-Str-σ1.0	0.9336 (0.0398)	0.2716 (0.1363)	0.888 (0.0468)	0.4776 (0.1503)
S-St1-σ1.5	0.6985 (0.4597)	0.014 (0.0804)	0.6944 (0.4572)	0.059 (0.1878)
S-St2-σ1.5	0.8608 (0.1871)	0.1929 (0.151)	0.7891 (0.182)	0.3591 (0.2084)
S-All-σ1.5	0.878 (0.1965)	0.1737 (0.1409)	0.8046 (0.1924)	0.3526 (0.206)
S-Str-σ1.5	0.9013 (0.1335)	0.1776 (0.1382)	0.8383 (0.1383)	0.3313 (0.1929)
