# Supplementary material for: Exploring the potential benefits of stratified false discovery rates for region-based testing of association with rare genetic variation
Source: Front Genet. 2014 Jan 29;5:11. doi: 10.3389/fgene.2014.00011 (PMC3905218; doi:10.3389/fgene.2014.00011)
Supplement: Table S1 — (A–D) True sensitivity (tSENS) and true FDR (tFDR) for different analytic strategies. Each table shows the tSENS and tFDR values for different p-value thresholds, ranging from 1e-08 (Table S1A) to 1e-03 (Table S1D). “m” is the mean over the 10 simulations, and “sd” is the standard deviation. Nomenclature follows Table 2. [file DataSheet1.ZIP › greenwood supp/10.3389.fgene.2014.00011 _Greenwood_Supplementary Table_3.PDF]

Tables S3.A - S3.D. True sensitivity (tSENS) and true FDR (tFDR) for different analytic strategies, H1-Corr≥ 0.75

Table S3.A. p-value threshold is 10^{-8}

	FDR				Sensitivity			
	H1		H1-Corr		H1		H1-Corr	
	m	sd	m	sd	m	sd	m.sen	sd.sen
N-All-σ0.5	0.9422	0.1706	0.8620	0.1879	0.1125	0.0957	0.4020	0.1952
N-St1-σ0.5	0.9557	0.1963	0.9192	0.1961	0.0468	0.0943	0.3015	0.2170
N-St2-σ0.5	0.8549	0.2323	0.7933	0.2225	0.1624	0.1371	0.3608	0.2056
N-All-σ1.0	0.8524	0.3058	0.7829	0.2971	0.0869	0.0808	0.3021	0.1967
N-St1-σ1.0	0.8824	0.3129	0.8458	0.3071	0.0364	0.0789	0.2148	0.2084
N-St2-σ1.0	0.7538	0.3334	0.6844	0.3113	0.1261	0.1142	0.2912	0.1916
N-All-σ1.5	0.7151	0.4072	0.6449	0.3870	0.0539	0.0578	0.1825	0.1784
N-St1-σ1.5	0.7140	0.4478	0.6876	0.4332	0.0214	0.0517	0.1304	0.1813
N-St2-σ1.5	0.6036	0.4181	0.5428	0.3895	0.0789	0.0854	0.1724	0.1682
P-All-σ0.5	0.9259	0.1950	0.8551	0.1993	0.1196	0.0942	0.3989	0.1915
P-St1-σ0.5	0.9556	0.1963	0.9186	0.1961	0.0464	0.0943	0.2920	0.2151
P-St2-σ0.5	0.8426	0.2247	0.7863	0.2123	0.1752	0.1372	0.3685	0.2028
P-All-σ1.0	0.8480	0.3053	0.7828	0.2974	0.0941	0.0843	0.2981	0.1964
P-St1-σ1.0	0.8815	0.3134	0.8425	0.3116	0.0364	0.0789	0.2126	0.2068
P-St2-σ1.0	0.7447	0.3376	0.6856	0.3122	0.1398	0.1257	0.2891	0.1925
P-All-σ1.5	0.7242	0.3967	0.6618	0.3771	0.0623	0.0624	0.1855	0.1805
P-St1-σ1.5	0.7241	0.4429	0.6955	0.4290	0.0211	0.0534	0.1299	0.1808
P-St2-σ1.5	0.6204	0.4056	0.5574	0.3799	0.0935	0.0940	0.1850	0.1711
S-All-σ0.5	0.7632	0.1585	0.6523	0.1463	0.1987	0.1249	0.3603	0.1568
S-St1-σ0.5	0.7468	0.4338	0.7372	0.4310	0.0125	0.0743	0.0385	0.1480
S-St2-σ0.5	0.7302	0.1694	0.6340	0.1544	0.2176	0.1354	0.3507	0.1545
S-All-σ1.0	0.6779	0.3270	0.5684	0.2924	0.0974	0.0906	0.1836	0.1332
S-St1-σ1.0	0.3460	0.4755	0.3435	0.4727	0.0050	0.0500	0.0150	0.1114
S-St2-σ1.0	0.6612	0.3304	0.5617	0.2933	0.1065	0.0987	0.1895	0.1349
S-All-σ1.5	0.5195	0.4156	0.4282	0.3598	0.0565	0.0701	0.1090	0.1083
S-St1-σ1.5	0.1700	0.3775	0.1635	0.3658	0.0000	0.0000	0.0167	0.1196
S-St2-σ1.5	0.5154	0.4139	0.4273	0.3590	0.0621	0.0771	0.1157	0.1113

Table S3.B. p-value threshold is 10^{-6}

	FDR				Sensitivity			
	H1		H1-Corr		H1		H1-Corr	
	m	sd	m	sd	m	sd	m.sen	sd.sen
N-All-σ0.5	0.9740	0.0354	0.8867	0.1207	0.1192	0.0964	0.4411	0.1835
N-St1-σ0.5	0.9864	0.0999	0.9425	0.1154	0.0500	0.0960	0.3520	0.2185
N-St2-σ0.5	0.8653	0.2022	0.8035	0.1944	0.1716	0.1366	0.3787	0.2036
N-All-σ1.0	0.8682	0.2941	0.8013	0.2806	0.0962	0.0865	0.3415	0.2030
N-St1-σ1.0	0.8947	0.3000	0.8580	0.2929	0.0377	0.0794	0.2434	0.2186
N-St2-σ1.0	0.7761	0.3205	0.7124	0.2976	0.1416	0.1270	0.3167	0.1952
N-All-σ1.5	0.7827	0.3659	0.7064	0.3514	0.0720	0.0725	0.2264	0.1984
N-St1-σ1.5	0.8254	0.3756	0.7933	0.3685	0.0273	0.0707	0.1624	0.1983
N-St2-σ1.5	0.6521	0.3972	0.5904	0.3696	0.1056	0.0990	0.2196	0.1871
P-All-σ0.5	0.9508	0.1411	0.8733	0.1567	0.1285	0.0953	0.4395	0.1838
P-St1-σ0.5	0.9763	0.1404	0.9337	0.1469	0.0483	0.0953	0.3420	0.2131
P-St2-σ0.5	0.8441	0.2225	0.7827	0.2113	0.1891	0.1395	0.3914	0.1993
P-All-σ1.0	0.8851	0.2662	0.8121	0.2605	0.1037	0.0883	0.3419	0.2012
P-St1-σ1.0	0.9047	0.2862	0.8674	0.2800	0.0377	0.0794	0.2388	0.2153
P-St2-σ1.0	0.7807	0.3078	0.7191	0.2863	0.1542	0.1309	0.3220	0.1976
P-All-σ1.5	0.7727	0.3677	0.7045	0.3532	0.0781	0.0757	0.2279	0.1987
P-St1-σ1.5	0.8254	0.3756	0.7934	0.3684	0.0266	0.0706	0.1615	0.1972
P-St2-σ1.5	0.6456	0.3964	0.5909	0.3702	0.1171	0.1067	0.2247	0.1936
S-All-σ0.5	0.8104	0.1168	0.7082	0.1159	0.2396	0.1307	0.4391	0.1553
S-St1-σ0.5	0.9375	0.2385	0.9223	0.2402	0.0125	0.0743	0.0650	0.1829
S-St2-σ0.5	0.7714	0.1271	0.6832	0.1239	0.2631	0.1403	0.4162	0.1481
S-All-σ1.0	0.6871	0.2826	0.5903	0.2513	0.1285	0.1077	0.2265	0.1457
S-St1-σ1.0	0.5143	0.4985	0.5068	0.4942	0.0100	0.0704	0.0267	0.1376
S-St2-σ1.0	0.6740	0.2821	0.5846	0.2516	0.1402	0.1182	0.2323	0.1498
S-All-σ1.5	0.5961	0.3816	0.5023	0.3403	0.0767	0.0774	0.1404	0.1192
S-St1-σ1.5	0.2200	0.4163	0.2135	0.4066	0.0000	0.0000	0.0167	0.1196
S-St2-σ1.5	0.5808	0.3813	0.4991	0.3423	0.0845	0.0852	0.1434	0.1194

Table S3.C. p-value threshold is 10^{-5}

	FDR				Sensitivity			
	H1		H1-Corr		H1		H1-Corr	
	m	sd	m	sd	m	sd	m.sen	sd.sen
N-All-σ0.5	0.9759	0.0312	0.9010	0.0943	0.1254	0.0958	0.4751	0.1649
N-St1-σ0.5	0.9970	0.0058	0.9613	0.0410	0.0507	0.0975	0.3836	0.2217
N-St2-σ0.5	0.8811	0.1592	0.8191	0.1553	0.1824	0.1362	0.3991	0.2034
N-All-σ1.0	0.9312	0.1941	0.8588	0.1962	0.1040	0.0887	0.3693	0.2033
N-St1-σ1.0	0.9552	0.1962	0.9177	0.1969	0.0401	0.0825	0.2669	0.2222
N-St2-σ1.0	0.7856	0.3096	0.7300	0.2887	0.1535	0.1322	0.3345	0.1998
N-All-σ1.5	0.8980	0.2394	0.8113	0.2525	0.0783	0.0762	0.2624	0.2001
N-St1-σ1.5	0.9350	0.2377	0.8973	0.2428	0.0314	0.0746	0.1833	0.2070
N-St2-σ1.5	0.7076	0.3738	0.6377	0.3435	0.1140	0.1044	0.2502	0.1933
P-All-σ0.5	0.9694	0.0445	0.8950	0.0970	0.1358	0.0961	0.4720	0.1700
P-St1-σ0.5	0.9968	0.0064	0.9594	0.0438	0.0493	0.0951	0.3694	0.2223
P-St2-σ0.5	0.8635	0.1724	0.7994	0.1686	0.2017	0.1403	0.4114	0.1978
P-All-σ1.0	0.9270	0.1858	0.8468	0.1954	0.1091	0.0895	0.3694	0.2002
P-St1-σ1.0	0.9553	0.1963	0.9097	0.2056	0.0391	0.0805	0.2649	0.2189
P-St2-σ1.0	0.8027	0.2882	0.7463	0.2700	0.1624	0.1336	0.3390	0.1994
P-All-σ1.5	0.9048	0.2223	0.8172	0.2432	0.0847	0.0802	0.2647	0.1998
P-St1-σ1.5	0.9449	0.2182	0.9067	0.2252	0.0314	0.0746	0.1846	0.2079
P-St2-σ1.5	0.6944	0.3737	0.6369	0.3449	0.1250	0.1120	0.2544	0.1994
S-All-σ0.5	0.8504	0.0811	0.7542	0.0801	0.2669	0.1299	0.5106	0.1468
S-St1-σ0.5	0.9857	0.1013	0.9601	0.1175	0.0262	0.1114	0.1284	0.2653
S-St2-σ0.5	0.8103	0.0963	0.7244	0.0898	0.2920	0.1384	0.4813	0.1384
S-All-σ1.0	0.7260	0.2322	0.6265	0.2097	0.1528	0.1124	0.2689	0.1488
S-St1-σ1.0	0.5767	0.4938	0.5678	0.4894	0.0100	0.0704	0.0307	0.1425
S-St2-σ1.0	0.7062	0.2337	0.6179	0.2111	0.1676	0.1231	0.2694	0.1511
S-All-σ1.5	0.6292	0.3447	0.5306	0.3011	0.0940	0.0856	0.1694	0.1269
S-St1-σ1.5	0.3200	0.4688	0.3082	0.4552	0.0000	0.0000	0.0225	0.1258
S-St2-σ1.5	0.6122	0.3456	0.5270	0.3053	0.1031	0.0933	0.1708	0.1276

Table S3.D. p-value threshold is 10^{-3}

	FDR				Sensitivity			
	H1		H1-Corr		H1		H1-Corr	
	m	sd	m	sd	m	sd	m.sen	sd.sen
N-All-σ0.5	0.9909	0.0060	0.9506	0.0339	0.1498	0.0993	0.6663	0.1111
N-St1-σ0.5	0.9986	0.0024	0.9774	0.0166	0.0603	0.1083	0.6053	0.1781
N-St2-σ0.5	0.9289	0.0638	0.8584	0.0848	0.2187	0.1398	0.4841	0.1731
N-All-σ1.0	0.9857	0.0117	0.9214	0.0445	0.1181	0.0962	0.5369	0.1333
N-St1-σ1.0	0.9976	0.0045	0.9672	0.0229	0.0466	0.0897	0.4370	0.2032
N-St2-σ1.0	0.9100	0.0969	0.8161	0.1335	0.1727	0.1385	0.4009	0.1877
N-All-σ1.5	0.9849	0.0139	0.9030	0.0535	0.0984	0.0885	0.4865	0.1357
N-St1-σ1.5	0.9970	0.0057	0.9589	0.0292	0.0418	0.0862	0.3913	0.1949
N-St2-σ1.5	0.9193	0.0921	0.8128	0.1338	0.1412	0.1232	0.3494	0.1987
P-All-σ0.5	0.9891	0.0068	0.9457	0.0381	0.1602	0.0969	0.6668	0.1018
P-St1-σ0.5	0.9985	0.0025	0.9753	0.0191	0.0578	0.1059	0.5925	0.1739
P-St2-σ0.5	0.9167	0.0718	0.8424	0.1086	0.2404	0.1407	0.5042	0.1692
P-All-σ1.0	0.9837	0.0130	0.9196	0.0453	0.1249	0.0983	0.5301	0.1324
P-St1-σ1.0	0.9975	0.0046	0.9670	0.0230	0.0459	0.0883	0.4213	0.2044
P-St2-σ1.0	0.8860	0.1458	0.8007	0.1608	0.1856	0.1450	0.4063	0.1856
P-All-σ1.5	0.9828	0.0147	0.9010	0.0555	0.1049	0.0897	0.4825	0.1318
P-St1-σ1.5	0.9970	0.0059	0.9586	0.0292	0.0408	0.0853	0.3855	0.1938
P-St2-σ1.5	0.8928	0.1393	0.8009	0.1592	0.1538	0.1288	0.3492	0.1985
S-All-σ0.5	0.9294	0.0334	0.8625	0.0400	0.3182	0.1398	0.6649	0.1147
S-St1-σ0.5	0.9977	0.0091	0.9795	0.0318	0.0312	0.1210	0.2073	0.3121
S-St2-σ0.5	0.9021	0.0462	0.8337	0.0504	0.3473	0.1452	0.6385	0.1285
S-All-σ1.0	0.8589	0.0852	0.7497	0.0818	0.2176	0.1224	0.4427	0.1292
S-St1-σ1.0	0.9630	0.1757	0.9456	0.1785	0.0147	0.0772	0.0866	0.2077
S-St2-σ1.0	0.8206	0.1064	0.7199	0.0976	0.2375	0.1319	0.4181	0.1320
S-All-σ1.5	0.8352	0.1371	0.7184	0.1308	0.1515	0.1201	0.3104	0.1670
S-St1-σ1.5	0.8871	0.3141	0.8764	0.3130	0.0100	0.0704	0.0475	0.1651
S-St2-σ1.5	0.8044	0.1519	0.7017	0.1424	0.1650	0.1311	0.2951	0.1615
