# Supplementary material for: Exploring the potential benefits of stratified false discovery rates for region-based testing of association with rare genetic variation
Source: Front Genet. 2014 Jan 29;5:11. doi: 10.3389/fgene.2014.00011 (PMC3905218; doi:10.3389/fgene.2014.00011)
Supplement: Table S1 — (A–D) True sensitivity (tSENS) and true FDR (tFDR) for different analytic strategies. Each table shows the tSENS and tFDR values for different p-value thresholds, ranging from 1e-08 (Table S1A) to 1e-03 (Table S1D). “m” is the mean over the 10 simulations, and “sd” is the standard deviation. Nomenclature follows Table 2. [file DataSheet1.ZIP › greenwood supp/10.3389.fgene.2014.00011 _Greenwood_Supplementary Table_4.PDF]

Tables S4.A - S4.I. Sensitivities and proportion of truly null window tests (tFDR) for different estimated values of FDR (H1-Corr≥ 0.75)

Table S4.A. The estimated FDR is 0.05 using the method: BH

	H1		H1-Corr	
	FDR	sensitivity	FDR	sensitivity
N-St1-σ0.5	0.9981 (0.0036)	0.0586 (0.112)	0.9681 (0.0377)	0.4842 (0.2468)
N-St2-σ0.5	0.9116 (0.1066)	0.2205 (0.1472)	0.8473 (0.1134)	0.4741 (0.2122)
N-All-σ0.5	0.9808 (0.0332)	0.1441 (0.1022)	0.9221 (0.0913)	0.5678 (0.1884)
N-Str-σ0.5	0.9797 (0.036)	0.1499 (0.1038)	0.9423 (0.0549)	0.4759 (0.1989)
N-St1-σ1.0	0.896 (0.3003)	0.0419 (0.0832)	0.8622 (0.2934)	0.2976 (0.2356)
N-St2-σ1.0	0.7982 (0.3062)	0.1696 (0.1381)	0.7458 (0.2878)	0.3698 (0.2159)
N-All-σ1.0	0.8753 (0.2949)	0.1101 (0.094)	0.8141 (0.2836)	0.3977 (0.2154)
N-Str-σ1.0	0.8745 (0.2946)	0.1143 (0.0943)	0.8349 (0.2845)	0.3384 (0.2019)
N-St1-σ1.5	0.8357 (0.3667)	0.0371 (0.0855)	0.8096 (0.3616)	0.2039 (0.227)
N-St2-σ1.5	0.7381 (0.36)	0.1377 (0.1197)	0.6759 (0.3351)	0.3081 (0.216)
N-All-σ1.5	0.8107 (0.3512)	0.089 (0.0869)	0.7451 (0.3401)	0.2929 (0.2238)
N-Str-σ1.5	0.8331 (0.3238)	0.0944 (0.0872)	0.7802 (0.3149)	0.2628 (0.1978)
P-St1-σ0.5	0.9879 (0.0999)	0.0567 (0.1058)	0.957 (0.1042)	0.4677 (0.2454)
P-St2-σ0.5	0.8996 (0.1182)	0.2388 (0.1456)	0.836 (0.1245)	0.4846 (0.2124)
P-All-σ0.5	0.9735 (0.0641)	0.1513 (0.101)	0.9131 (0.1043)	0.5577 (0.1898)
P-Str-σ0.5	0.9712 (0.0745)	0.159 (0.0999)	0.9322 (0.0822)	0.4741 (0.1975)
P-St1-σ1.0	0.8959 (0.3003)	0.0413 (0.0833)	0.8612 (0.2939)	0.2874 (0.2317)
P-St2-σ1.0	0.8181 (0.2665)	0.1828 (0.1463)	0.7623 (0.2524)	0.3827 (0.2173)
P-All-σ1.0	0.8826 (0.2814)	0.1144 (0.0956)	0.8207 (0.2718)	0.3943 (0.2135)
P-Str-σ1.0	0.8974 (0.2439)	0.1215 (0.098)	0.8549 (0.2395)	0.3409 (0.2019)
P-St1-σ1.5	0.8158 (0.3843)	0.0364 (0.0844)	0.7963 (0.3756)	0.2002 (0.2261)
P-St2-σ1.5	0.7341 (0.3576)	0.1464 (0.1239)	0.6729 (0.3331)	0.3122 (0.2161)
P-All-σ1.5	0.7852 (0.3673)	0.0933 (0.0876)	0.7352 (0.3488)	0.2913 (0.2227)
P-Str-σ1.5	0.8191 (0.3345)	0.0988 (0.0879)	0.7727 (0.3219)	0.2631 (0.1974)
S-St1-σ0.5	0.9977 (0.0091)	0.0312 (0.121)	0.9773 (0.0376)	0.2073 (0.3121)
S-St2-σ0.5	0.9014 (0.049)	0.3461 (0.1457)	0.8331 (0.0538)	0.6384 (0.1361)
S-All-σ0.5	0.9271 (0.0378)	0.3154 (0.141)	0.8588 (0.046)	0.6609 (0.1217)
S-Str-σ0.5	0.9274 (0.0369)	0.3173 (0.1405)	0.8699 (0.0445)	0.6062 (0.1268)
S-St1-σ1.0	0.6864 (0.4629)	0.0125 (0.0743)	0.6729 (0.4571)	0.0525 (0.1717)
S-St2-σ1.0	0.7772 (0.1511)	0.2159 (0.1321)	0.6773 (0.1426)	0.3628 (0.1526)
S-All-σ1.0	0.7967 (0.159)	0.1933 (0.1177)	0.6865 (0.1477)	0.3634 (0.1594)
S-Str-σ1.0	0.8019 (0.15)	0.1968 (0.1219)	0.7096 (0.1453)	0.3346 (0.1437)
S-St1-σ1.5	0.4586 (0.4996)	0.005 (0.05)	0.4496 (0.4919)	0.0275 (0.1346)
S-St2-σ1.5	0.6643 (0.3306)	0.1218 (0.1105)	0.5818 (0.2952)	0.209 (0.1473)
S-All-σ1.5	0.6723 (0.3325)	0.1104 (0.1005)	0.5756 (0.2942)	0.2115 (0.1539)
S-Str-σ1.5	0.6925 (0.3203)	0.1114 (0.1014)	0.6144 (0.2913)	0.1917 (0.1377)

Table S4.B. The estimated FDR is 0.25 using the method: BH

	H1		H1-Corr	
	FDR	sensitivity	FDR	sensitivity
N-St1-σ0.5	0.999 (0.0016)	0.0736 (0.1174)	0.9797 (0.0282)	0.6675 (0.2279)
N-St2-σ0.5	0.9455 (0.0623)	0.2636 (0.1524)	0.89 (0.0822)	0.5866 (0.2124)
N-All-σ0.5	0.9918 (0.0112)	0.168 (0.1156)	0.9535 (0.0596)	0.7197 (0.1681)
N-Str-σ0.5	0.9912 (0.0105)	0.1818 (0.1139)	0.9663 (0.033)	0.6208 (0.194)
N-St1-σ1.0	0.9375 (0.2381)	0.0492 (0.0968)	0.9086 (0.2335)	0.3938 (0.2637)
N-St2-σ1.0	0.8502 (0.2601)	0.1877 (0.1409)	0.7902 (0.2513)	0.4443 (0.2403)
N-All-σ1.0	0.9233 (0.2356)	0.1193 (0.0998)	0.8658 (0.2327)	0.4883 (0.2325)
N-Str-σ1.0	0.9329 (0.2025)	0.1276 (0.1009)	0.8914 (0.2035)	0.4214 (0.2271)
N-St1-σ1.5	0.9169 (0.2718)	0.042 (0.0917)	0.8809 (0.2721)	0.2906 (0.2505)
N-St2-σ1.5	0.7862 (0.3356)	0.153 (0.1292)	0.7232 (0.3187)	0.3735 (0.2411)
N-All-σ1.5	0.8899 (0.2706)	0.0977 (0.0907)	0.8189 (0.2724)	0.3854 (0.2422)
N-Str-σ1.5	0.8912 (0.2693)	0.1053 (0.0937)	0.837 (0.2695)	0.3361 (0.2235)
P-St1-σ0.5	0.9989 (0.0019)	0.067 (0.1117)	0.977 (0.0304)	0.6375 (0.236)
P-St2-σ0.5	0.9344 (0.0718)	0.288 (0.1565)	0.8752 (0.1012)	0.5976 (0.2068)
P-All-σ0.5	0.9895 (0.0149)	0.1754 (0.1078)	0.9478 (0.0613)	0.7055 (0.1666)
P-Str-σ0.5	0.988 (0.0164)	0.1924 (0.112)	0.9595 (0.0441)	0.6141 (0.1932)
P-St1-σ1.0	0.9373 (0.2381)	0.0482 (0.0944)	0.9057 (0.2342)	0.3843 (0.259)
P-St2-σ1.0	0.8685 (0.2233)	0.2045 (0.1497)	0.8059 (0.2225)	0.4588 (0.2424)
P-All-σ1.0	0.9388 (0.1861)	0.1264 (0.1028)	0.872 (0.1991)	0.4871 (0.2273)
P-Str-σ1.0	0.9391 (0.1806)	0.1363 (0.1041)	0.8946 (0.1856)	0.4254 (0.2229)
P-St1-σ1.5	0.8869 (0.3134)	0.0408 (0.0896)	0.8463 (0.31)	0.2905 (0.2515)
P-St2-σ1.5	0.774 (0.3418)	0.1637 (0.135)	0.7111 (0.3208)	0.3848 (0.2461)
P-All-σ1.5	0.8576 (0.3089)	0.1018 (0.0918)	0.7897 (0.3029)	0.3829 (0.241)
P-Str-σ1.5	0.8764 (0.2839)	0.1111 (0.0961)	0.8187 (0.2806)	0.342 (0.2245)
S-St1-σ0.5	0.9979 (0.0068)	0.0537 (0.1832)	0.9846 (0.0209)	0.2743 (0.3505)
S-St2-σ0.5	0.9365 (0.0285)	0.3892 (0.1445)	0.8871 (0.0359)	0.7228 (0.1144)
S-All-σ0.5	0.9538 (0.0208)	0.3554 (0.1409)	0.9068 (0.0293)	0.7426 (0.1068)
S-Str-σ0.5	0.9538 (0.0207)	0.3573 (0.1401)	0.9134 (0.0275)	0.689 (0.1125)
S-St1-σ1.0	0.8836 (0.3149)	0.0181 (0.0835)	0.8657 (0.3115)	0.1196 (0.2373)
S-St2-σ1.0	0.8607 (0.0946)	0.256 (0.146)	0.767 (0.1014)	0.4704 (0.1647)
S-All-σ1.0	0.8813 (0.0892)	0.2325 (0.135)	0.7804 (0.0999)	0.4845 (0.1612)
S-Str-σ1.0	0.887 (0.0873)	0.2347 (0.1354)	0.8071 (0.0922)	0.4413 (0.1575)
S-St1-σ1.5	0.677 (0.4671)	0.014 (0.0804)	0.6686 (0.4626)	0.0482 (0.1664)
S-St2-σ1.5	0.7794 (0.2346)	0.1723 (0.1438)	0.6867 (0.2139)	0.313 (0.1972)
S-All-σ1.5	0.7873 (0.2457)	0.1503 (0.1298)	0.6841 (0.2229)	0.3046 (0.1981)
S-Str-σ1.5	0.8177 (0.1986)	0.1587 (0.1315)	0.7336 (0.1861)	0.2886 (0.1831)

Table S4.C. The estimated FDR is 0.5 using the method: BH

	H1		H1-Corr	
	FDR	sensitivity	FDR	sensitivity
N-St1-σ0.5	0.9994 (7e-04)	0.1113 (0.1375)	0.9893 (0.0155)	0.8203 (0.1602)
N-St2-σ0.5	0.9668 (0.0508)	0.317 (0.1595)	0.9259 (0.0729)	0.703 (0.1812)
N-All-σ0.5	0.9965 (0.0031)	0.213 (0.1322)	0.9783 (0.03)	0.8442 (0.1166)
N-Str-σ0.5	0.9961 (0.0033)	0.2291 (0.1295)	0.982 (0.0206)	0.753 (0.1456)
N-St1-σ1.0	0.9486 (0.2187)	0.0566 (0.1109)	0.9243 (0.2154)	0.5186 (0.2727)
N-St2-σ1.0	0.9042 (0.207)	0.2159 (0.1591)	0.8357 (0.2174)	0.5503 (0.2576)
N-All-σ1.0	0.9397 (0.2171)	0.1325 (0.1084)	0.8918 (0.2169)	0.5995 (0.2455)
N-Str-σ1.0	0.9568 (0.1707)	0.1475 (0.1153)	0.9192 (0.1735)	0.5379 (0.2408)
N-St1-σ1.5	0.9279 (0.2559)	0.0479 (0.0965)	0.8961 (0.2583)	0.3866 (0.2849)
N-St2-σ1.5	0.851 (0.2941)	0.1687 (0.1364)	0.7896 (0.2803)	0.4664 (0.2569)
N-All-σ1.5	0.9114 (0.2551)	0.1062 (0.0964)	0.8469 (0.2604)	0.4717 (0.2764)
N-Str-σ1.5	0.925 (0.236)	0.1175 (0.1016)	0.8814 (0.2322)	0.4294 (0.2515)
P-St1-σ0.5	0.9994 (9e-04)	0.0993 (0.1333)	0.9867 (0.0192)	0.7901 (0.1815)
P-St2-σ0.5	0.9606 (0.0447)	0.3566 (0.1741)	0.9169 (0.0789)	0.7109 (0.1794)
P-All-σ0.5	0.9952 (0.0044)	0.2224 (0.1327)	0.9727 (0.0395)	0.8321 (0.1243)
P-Str-σ0.5	0.9945 (0.0046)	0.2454 (0.1325)	0.977 (0.0291)	0.7453 (0.1529)
P-St1-σ1.0	0.9685 (0.1712)	0.054 (0.1083)	0.9375 (0.1805)	0.4946 (0.2703)
P-St2-σ1.0	0.907 (0.1866)	0.2345 (0.1609)	0.8391 (0.1972)	0.5588 (0.2485)
P-All-σ1.0	0.9705 (0.1133)	0.1387 (0.1108)	0.9173 (0.1361)	0.5891 (0.2405)
P-Str-σ1.0	0.9738 (0.1015)	0.1561 (0.1131)	0.9308 (0.1232)	0.5312 (0.2364)
P-St1-σ1.5	0.9559 (0.1972)	0.0464 (0.0935)	0.9241 (0.2056)	0.3826 (0.2825)
P-St2-σ1.5	0.8569 (0.28)	0.1831 (0.1426)	0.7915 (0.2688)	0.4743 (0.2493)
P-All-σ1.5	0.9427 (0.1963)	0.1115 (0.0981)	0.8683 (0.2189)	0.4727 (0.2687)
P-Str-σ1.5	0.941 (0.1956)	0.1246 (0.1012)	0.8938 (0.1966)	0.4327 (0.2438)
S-St1-σ0.5	0.9977 (0.0065)	0.0701 (0.1991)	0.9872 (0.0168)	0.3281 (0.3787)
S-St2-σ0.5	0.9507 (0.0202)	0.4287 (0.1358)	0.9135 (0.0285)	0.7713 (0.1061)
S-All-σ0.5	0.9644 (0.0137)	0.3916 (0.1303)	0.9291 (0.0218)	0.7918 (0.0891)
S-Str-σ0.5	0.9643 (0.014)	0.3946 (0.1309)	0.9339 (0.0211)	0.7373 (0.1025)
S-St1-σ1.0	0.976 (0.1415)	0.0206 (0.0866)	0.9587 (0.1449)	0.1707 (0.2827)
S-St2-σ1.0	0.9154 (0.0483)	0.2984 (0.1475)	0.842 (0.0585)	0.602 (0.1586)
S-All-σ1.0	0.936 (0.0374)	0.2696 (0.1403)	0.8631 (0.0529)	0.6141 (0.1498)
S-Str-σ1.0	0.9353 (0.0387)	0.2731 (0.1371)	0.874 (0.0491)	0.5671 (0.1492)
S-St1-σ1.5	0.8485 (0.3583)	0.014 (0.0804)	0.8352 (0.3543)	0.0984 (0.2194)
S-St2-σ1.5	0.8761 (0.1424)	0.1984 (0.1542)	0.7833 (0.1438)	0.4222 (0.2227)
S-All-σ1.5	0.8806 (0.1796)	0.1768 (0.1393)	0.7805 (0.1717)	0.427 (0.226)
S-Str-σ1.5	0.9011 (0.1335)	0.1824 (0.1409)	0.8201 (0.136)	0.3943 (0.2066)

Table S4.D. The estimated FDR is 0.05 using the method: BUM

	H1		H1-Corr	
	FDR	sensitivity	FDR	sensitivity
N-St1-σ0.5	0.9882 (0.0999)	0.0586 (0.112)	0.9589 (0.1031)	0.4997 (0.2471)
N-St2-σ0.5	0.9114 (0.1064)	0.22 (0.1471)	0.8477 (0.1108)	0.472 (0.2117)
N-All-σ0.5	0.9824 (0.0299)	0.1442 (0.1029)	0.9245 (0.0882)	0.5795 (0.1867)
N-Str-σ0.5	0.9799 (0.0416)	0.1496 (0.1037)	0.9442 (0.0559)	0.4811 (0.1986)
N-St1-σ1.0	0.8861 (0.3132)	0.0419 (0.0832)	0.8557 (0.3053)	0.2986 (0.238)
N-St2-σ1.0	0.7882 (0.3159)	0.169 (0.138)	0.7358 (0.2963)	0.3698 (0.2159)
N-All-σ1.0	0.8654 (0.3074)	0.1104 (0.0948)	0.8079 (0.2945)	0.4002 (0.2168)
N-Str-σ1.0	0.8645 (0.3071)	0.1139 (0.0941)	0.8276 (0.2965)	0.3393 (0.2026)
N-St1-σ1.5	0.7458 (0.4329)	0.0364 (0.0844)	0.7251 (0.4213)	0.2052 (0.226)
N-St2-σ1.5	0.7187 (0.373)	0.1374 (0.1213)	0.6633 (0.3455)	0.3072 (0.2148)
N-All-σ1.5	0.7459 (0.4031)	0.0882 (0.0867)	0.7015 (0.3817)	0.2948 (0.2242)
N-Str-σ1.5	0.7794 (0.3785)	0.0938 (0.0875)	0.7382 (0.3603)	0.2625 (0.1976)
P-St1-σ0.5	0.9779 (0.1405)	0.0571 (0.1066)	0.9451 (0.1468)	0.4724 (0.2438)
P-St2-σ0.5	0.8987 (0.1189)	0.2391 (0.145)	0.8332 (0.1263)	0.487 (0.2118)
P-All-σ0.5	0.9696 (0.1065)	0.1518 (0.1008)	0.9108 (0.1301)	0.5655 (0.1852)
P-Str-σ0.5	0.9704 (0.0734)	0.1593 (0.0999)	0.9319 (0.087)	0.4783 (0.1988)
P-St1-σ1.0	0.8861 (0.3132)	0.0409 (0.0834)	0.8542 (0.3057)	0.2898 (0.2313)
P-St2-σ1.0	0.7928 (0.3066)	0.1804 (0.1487)	0.7396 (0.2879)	0.38 (0.2224)
P-All-σ1.0	0.8628 (0.307)	0.1143 (0.0955)	0.8045 (0.2941)	0.3961 (0.2137)
P-Str-σ1.0	0.872 (0.2944)	0.1198 (0.0996)	0.8339 (0.2848)	0.3406 (0.2045)
P-St1-σ1.5	0.7457 (0.4328)	0.0364 (0.0844)	0.7219 (0.4201)	0.204 (0.2238)
P-St2-σ1.5	0.7157 (0.3697)	0.1463 (0.1253)	0.6604 (0.3422)	0.3136 (0.2174)
P-All-σ1.5	0.7398 (0.4019)	0.0933 (0.0873)	0.6972 (0.3795)	0.2923 (0.2209)
P-Str-σ1.5	0.7797 (0.3723)	0.0986 (0.0888)	0.7382 (0.3539)	0.2655 (0.1973)
S-St1-σ0.5	0.9977 (0.0092)	0.0312 (0.121)	0.9774 (0.0381)	0.2039 (0.3125)
S-St2-σ0.5	0.9006 (0.0488)	0.3446 (0.1462)	0.832 (0.0537)	0.6332 (0.1352)
S-All-σ0.5	0.9271 (0.0371)	0.3128 (0.1397)	0.8582 (0.0456)	0.6575 (0.1211)
S-Str-σ0.5	0.9258 (0.0375)	0.3159 (0.1407)	0.8676 (0.0451)	0.6006 (0.1263)
S-St1-σ1.0	0.6664 (0.4706)	0.0125 (0.0743)	0.6544 (0.4655)	0.0458 (0.1601)
S-St2-σ1.0	0.7767 (0.1496)	0.2165 (0.1337)	0.679 (0.1414)	0.361 (0.1562)
S-All-σ1.0	0.7972 (0.1572)	0.1946 (0.1202)	0.689 (0.1467)	0.3659 (0.1608)
S-Str-σ1.0	0.7981 (0.1487)	0.1975 (0.1234)	0.7078 (0.1437)	0.332 (0.1484)
S-St1-σ1.5	0.368 (0.483)	0.005 (0.05)	0.3591 (0.4734)	0.0275 (0.1346)
S-St2-σ1.5	0.635 (0.3385)	0.1247 (0.1107)	0.5525 (0.2989)	0.2126 (0.1466)
S-All-σ1.5	0.6572 (0.3435)	0.1111 (0.0999)	0.5585 (0.3016)	0.2126 (0.1534)
S-Str-σ1.5	0.6541 (0.3372)	0.1142 (0.1017)	0.5754 (0.3026)	0.1951 (0.1371)

Table S4.E. The estimated FDR is 0.25 using the method: BUM

	H1		H1-Corr	
	FDR	sensitivity	FDR	sensitivity
N-St1-σ0.5	0.9989 (0.0019)	0.0696 (0.1167)	0.9789 (0.0276)	0.65 (0.2207)
N-St2-σ0.5	0.9402 (0.0673)	0.2561 (0.1495)	0.8786 (0.0908)	0.5737 (0.2104)
N-All-σ0.5	0.9917 (0.0085)	0.162 (0.1099)	0.953 (0.0553)	0.7081 (0.1542)
N-Str-σ0.5	0.9911 (0.0085)	0.1757 (0.1106)	0.9646 (0.0346)	0.6047 (0.1819)
N-St1-σ1.0	0.9074 (0.2869)	0.0492 (0.0968)	0.8801 (0.2801)	0.3848 (0.2652)
N-St2-σ1.0	0.834 (0.2836)	0.1859 (0.1412)	0.7807 (0.2706)	0.4398 (0.2469)
N-All-σ1.0	0.8829 (0.2967)	0.1189 (0.0994)	0.8303 (0.2864)	0.4797 (0.2379)
N-Str-σ1.0	0.9067 (0.2557)	0.1267 (0.1008)	0.8714 (0.2511)	0.4154 (0.2322)
N-St1-σ1.5	0.7769 (0.4147)	0.0408 (0.0896)	0.751 (0.403)	0.2788 (0.2471)
N-St2-σ1.5	0.7717 (0.3493)	0.1518 (0.1286)	0.7152 (0.3289)	0.377 (0.2446)
N-All-σ1.5	0.7903 (0.3769)	0.0964 (0.0908)	0.738 (0.3584)	0.3693 (0.2474)
N-Str-σ1.5	0.8255 (0.3507)	0.1044 (0.0933)	0.7827 (0.3376)	0.3331 (0.2246)
P-St1-σ0.5	0.9888 (0.0999)	0.0635 (0.1109)	0.9694 (0.0996)	0.6153 (0.2358)
P-St2-σ0.5	0.9307 (0.0649)	0.2824 (0.156)	0.8631 (0.112)	0.5799 (0.197)
P-All-σ0.5	0.9895 (0.0115)	0.1693 (0.1061)	0.9462 (0.059)	0.6922 (0.1603)
P-Str-σ0.5	0.9864 (0.0233)	0.1877 (0.1116)	0.9528 (0.0754)	0.5935 (0.1815)
P-St1-σ1.0	0.9073 (0.2868)	0.0482 (0.0944)	0.8787 (0.2803)	0.3695 (0.2619)
P-St2-σ1.0	0.8349 (0.2728)	0.2019 (0.1502)	0.7797 (0.2609)	0.4483 (0.2448)
P-All-σ1.0	0.8809 (0.2962)	0.1252 (0.1041)	0.8268 (0.2863)	0.4754 (0.2364)
P-Str-σ1.0	0.9119 (0.2378)	0.1349 (0.1046)	0.8739 (0.2355)	0.4133 (0.2276)
P-St1-σ1.5	0.7668 (0.4212)	0.0414 (0.0895)	0.7409 (0.4088)	0.2751 (0.2482)
P-St2-σ1.5	0.7554 (0.3579)	0.1644 (0.1365)	0.7022 (0.3368)	0.3819 (0.2502)
P-All-σ1.5	0.7801 (0.3839)	0.1006 (0.0932)	0.7293 (0.3632)	0.3704 (0.2466)
P-Str-σ1.5	0.8159 (0.3537)	0.1116 (0.0968)	0.7745 (0.3402)	0.3332 (0.2265)
S-St1-σ0.5	0.9978 (0.0074)	0.0537 (0.1832)	0.9836 (0.0226)	0.2709 (0.3515)
S-St2-σ0.5	0.9333 (0.03)	0.3808 (0.1433)	0.8811 (0.0378)	0.7114 (0.1206)
S-All-σ0.5	0.9517 (0.0217)	0.3492 (0.1407)	0.9024 (0.0299)	0.7312 (0.1034)
S-Str-σ0.5	0.9512 (0.0221)	0.3498 (0.1401)	0.9083 (0.0294)	0.6785 (0.118)
S-St1-σ1.0	0.7776 (0.4153)	0.0125 (0.0743)	0.7614 (0.4089)	0.0981 (0.2193)
S-St2-σ1.0	0.8453 (0.0999)	0.2543 (0.1444)	0.7521 (0.1032)	0.4562 (0.161)
S-All-σ1.0	0.8716 (0.0916)	0.2286 (0.1321)	0.7687 (0.1002)	0.4705 (0.1569)
S-Str-σ1.0	0.8691 (0.0941)	0.2325 (0.1349)	0.7852 (0.1022)	0.4254 (0.1569)
S-St1-σ1.5	0.5074 (0.5002)	0.01 (0.0704)	0.4985 (0.4928)	0.0442 (0.1626)
S-St2-σ1.5	0.7419 (0.2763)	0.1669 (0.1462)	0.6497 (0.2489)	0.3004 (0.2003)
S-All-σ1.5	0.77 (0.2659)	0.1456 (0.124)	0.6639 (0.2395)	0.2934 (0.1902)
S-Str-σ1.5	0.7796 (0.253)	0.153 (0.1341)	0.6932 (0.2349)	0.2764 (0.1862)

Table S4.F. The estimated FDR is 0.5 using the method: BUM

	H1		H1-Corr	
	FDR	sensitivity	FDR	sensitivity
N-St1-σ0.5	0.9992 (0.0011)	0.0896 (0.123)	0.9868 (0.0165)	0.7753 (0.1773)
N-St2-σ0.5	0.9589 (0.0566)	0.2923 (0.1522)	0.9107 (0.0763)	0.6636 (0.1879)
N-All-σ0.5	0.9953 (0.004)	0.1889 (0.1193)	0.9731 (0.0301)	0.8033 (0.1243)
N-Str-σ0.5	0.9948 (0.0042)	0.2057 (0.1177)	0.9779 (0.0209)	0.7101 (0.1446)
N-St1-σ1.0	0.9184 (0.2722)	0.0526 (0.1004)	0.8969 (0.2669)	0.4852 (0.2769)
N-St2-σ1.0	0.8636 (0.2688)	0.2123 (0.156)	0.8101 (0.261)	0.5277 (0.2719)
N-All-σ1.0	0.9094 (0.2698)	0.1278 (0.1049)	0.8629 (0.2636)	0.5716 (0.2466)
N-Str-σ1.0	0.9218 (0.2398)	0.1434 (0.1108)	0.8897 (0.2375)	0.5104 (0.2533)
N-St1-σ1.5	0.7979 (0.401)	0.0478 (0.0987)	0.7764 (0.391)	0.3738 (0.2894)
N-St2-σ1.5	0.839 (0.3073)	0.1709 (0.14)	0.7767 (0.2942)	0.4672 (0.2642)
N-All-σ1.5	0.8133 (0.3718)	0.1058 (0.0964)	0.7625 (0.3597)	0.4591 (0.2854)
N-Str-σ1.5	0.8798 (0.2994)	0.1184 (0.1034)	0.8318 (0.2938)	0.4237 (0.2523)
P-St1-σ0.5	0.9891 (0.0999)	0.0814 (0.1213)	0.9757 (0.0994)	0.741 (0.1992)
P-St2-σ0.5	0.9508 (0.052)	0.3293 (0.1624)	0.8999 (0.0919)	0.6648 (0.1809)
P-All-σ0.5	0.9937 (0.0055)	0.1975 (0.1177)	0.9667 (0.0396)	0.7905 (0.1189)
P-Str-σ0.5	0.9905 (0.0283)	0.2219 (0.1205)	0.9689 (0.0648)	0.6962 (0.1479)
P-St1-σ1.0	0.9183 (0.2722)	0.0521 (0.1053)	0.8945 (0.267)	0.4694 (0.2774)
P-St2-σ1.0	0.8831 (0.2361)	0.2319 (0.1623)	0.8194 (0.2355)	0.543 (0.2646)
P-All-σ1.0	0.918 (0.2536)	0.1329 (0.1096)	0.87 (0.2491)	0.5584 (0.2475)
P-Str-σ1.0	0.9424 (0.1954)	0.1536 (0.1126)	0.9023 (0.1999)	0.5111 (0.247)
P-St1-σ1.5	0.7879 (0.4083)	0.0455 (0.0963)	0.7646 (0.3974)	0.3721 (0.2897)
P-St2-σ1.5	0.8358 (0.3048)	0.185 (0.1434)	0.7787 (0.2921)	0.4655 (0.2631)
P-All-σ1.5	0.8028 (0.3795)	0.1097 (0.0984)	0.7602 (0.3634)	0.4528 (0.2835)
P-Str-σ1.5	0.8833 (0.2863)	0.1248 (0.1023)	0.8385 (0.2812)	0.4213 (0.2555)
S-St1-σ0.5	0.9971 (0.0074)	0.0776 (0.2042)	0.9867 (0.0181)	0.3251 (0.3793)
S-St2-σ0.5	0.9488 (0.0211)	0.4248 (0.1348)	0.9104 (0.0282)	0.7673 (0.1067)
S-All-σ0.5	0.9632 (0.0144)	0.3896 (0.1311)	0.927 (0.0221)	0.7875 (0.0878)
S-Str-σ0.5	0.9628 (0.0148)	0.393 (0.1305)	0.9316 (0.0214)	0.7339 (0.1026)
S-St1-σ1.0	0.8683 (0.3375)	0.0158 (0.0809)	0.8553 (0.3339)	0.1321 (0.2596)
S-St2-σ1.0	0.9012 (0.0572)	0.2841 (0.1477)	0.8213 (0.0677)	0.5596 (0.1594)
S-All-σ1.0	0.9209 (0.0497)	0.2533 (0.1381)	0.8372 (0.0647)	0.5715 (0.1535)
S-Str-σ1.0	0.9208 (0.051)	0.2591 (0.1383)	0.8522 (0.0608)	0.5223 (0.1521)
S-St1-σ1.5	0.5883 (0.493)	0.014 (0.0804)	0.5786 (0.4859)	0.061 (0.1759)
S-St2-σ1.5	0.8266 (0.2211)	0.1899 (0.156)	0.729 (0.2086)	0.3844 (0.2146)
S-All-σ1.5	0.8405 (0.2315)	0.1667 (0.1413)	0.7401 (0.2191)	0.3865 (0.2271)
S-Str-σ1.5	0.8607 (0.1863)	0.1746 (0.1425)	0.7701 (0.1847)	0.3547 (0.1992)

Table S4.G. The estimated FDR is 0.05 using the method: fdrtool

	H1		H1-Corr	
	FDR	sensitivity	FDR	sensitivity
N-St1-σ0.5	0.9982 (0.0035)	0.0586 (0.112)	0.9685 (0.0373)	0.4863 (0.2461)
N-St2-σ0.5	0.913 (0.1059)	0.2212 (0.1469)	0.848 (0.1136)	0.4781 (0.2099)
N-All-σ0.5	0.9815 (0.0302)	0.1449 (0.1022)	0.9224 (0.0916)	0.5753 (0.1898)
N-Str-σ0.5	0.98 (0.036)	0.1503 (0.1035)	0.9429 (0.0546)	0.479 (0.1981)
N-St1-σ1.0	0.8961 (0.3003)	0.0419 (0.0832)	0.8626 (0.2936)	0.2963 (0.237)
N-St2-σ1.0	0.7963 (0.3129)	0.1686 (0.1397)	0.7435 (0.2942)	0.3707 (0.2215)
N-All-σ1.0	0.8754 (0.2949)	0.1098 (0.0938)	0.8141 (0.2835)	0.3999 (0.2163)
N-Str-σ1.0	0.876 (0.2949)	0.1136 (0.0954)	0.8367 (0.285)	0.3388 (0.2063)
N-St1-σ1.5	0.7859 (0.4074)	0.0359 (0.0851)	0.7656 (0.3975)	0.2022 (0.2282)
N-St2-σ1.5	0.7346 (0.3662)	0.1364 (0.1208)	0.6679 (0.3392)	0.309 (0.2168)
N-All-σ1.5	0.7733 (0.3837)	0.0884 (0.0873)	0.7187 (0.3645)	0.2949 (0.2251)
N-Str-σ1.5	0.8061 (0.3528)	0.093 (0.0879)	0.7566 (0.338)	0.2623 (0.2008)
P-St1-σ0.5	0.9879 (0.0999)	0.0567 (0.1058)	0.9573 (0.1041)	0.4726 (0.2458)
P-St2-σ0.5	0.8998 (0.1195)	0.2395 (0.1453)	0.8359 (0.127)	0.4876 (0.2122)
P-All-σ0.5	0.9746 (0.0603)	0.1516 (0.1011)	0.9148 (0.1001)	0.5612 (0.1911)
P-Str-σ0.5	0.9715 (0.0745)	0.1593 (0.0997)	0.9327 (0.0823)	0.4781 (0.1982)
P-St1-σ1.0	0.896 (0.3003)	0.0413 (0.0833)	0.8611 (0.2941)	0.2871 (0.2324)
P-St2-σ1.0	0.8083 (0.2869)	0.1819 (0.1477)	0.7563 (0.2707)	0.3806 (0.2218)
P-All-σ1.0	0.8726 (0.2946)	0.1153 (0.0966)	0.8128 (0.2832)	0.3925 (0.2137)
P-Str-σ1.0	0.8968 (0.2543)	0.1211 (0.0989)	0.8565 (0.247)	0.3399 (0.2043)
P-St1-σ1.5	0.7661 (0.4209)	0.0351 (0.084)	0.7468 (0.4108)	0.198 (0.2275)
P-St2-σ1.5	0.7305 (0.3637)	0.1454 (0.1251)	0.665 (0.3369)	0.3149 (0.2173)
P-All-σ1.5	0.7611 (0.3902)	0.0923 (0.0883)	0.7053 (0.3705)	0.2938 (0.2219)
P-Str-σ1.5	0.7994 (0.3549)	0.0975 (0.0885)	0.7506 (0.3394)	0.2637 (0.1994)
S-St1-σ0.5	0.9974 (0.0102)	0.0312 (0.121)	0.9753 (0.0401)	0.2033 (0.3082)
S-St2-σ0.5	0.8908 (0.0523)	0.3372 (0.1448)	0.8188 (0.0553)	0.6123 (0.1358)
S-All-σ0.5	0.9196 (0.0395)	0.3088 (0.1388)	0.8463 (0.0451)	0.6422 (0.1193)
S-Str-σ0.5	0.9198 (0.0395)	0.3088 (0.1388)	0.8592 (0.0457)	0.5813 (0.1263)
S-St1-σ1.0	0.6664 (0.4706)	0.0125 (0.0743)	0.6551 (0.4654)	0.0492 (0.1694)
S-St2-σ1.0	0.7781 (0.1504)	0.2163 (0.1325)	0.6781 (0.1428)	0.3638 (0.1526)
S-All-σ1.0	0.7987 (0.1583)	0.1933 (0.1177)	0.6884 (0.1476)	0.3657 (0.1604)
S-Str-σ1.0	0.8015 (0.1496)	0.1972 (0.1222)	0.7093 (0.1452)	0.3348 (0.1437)
S-St1-σ1.5	0.3886 (0.4886)	0.005 (0.05)	0.3796 (0.4795)	0.0275 (0.1346)
S-St2-σ1.5	0.6582 (0.3301)	0.1221 (0.1101)	0.5781 (0.2961)	0.2075 (0.1475)
S-All-σ1.5	0.6725 (0.3325)	0.1104 (0.1005)	0.5759 (0.2942)	0.2115 (0.1539)
S-Str-σ1.5	0.6783 (0.3274)	0.1118 (0.1011)	0.6025 (0.2983)	0.1907 (0.1382)

Table S4.H. The estimated FDR is 0.25 using the method: fdrtool

	H1		H1-Corr	
	FDR	sensitivity	FDR	sensitivity
N-St1-σ0.5	0.9991 (0.0015)	0.0744 (0.118)	0.9802 (0.0281)	0.6781 (0.2259)
N-St2-σ0.5	0.9492 (0.0593)	0.2673 (0.1537)	0.8952 (0.0792)	0.6009 (0.212)
N-All-σ0.5	0.9923 (0.01)	0.1711 (0.117)	0.9552 (0.0583)	0.7302 (0.1668)
N-Str-σ0.5	0.9917 (0.0098)	0.1845 (0.115)	0.967 (0.0332)	0.6336 (0.1922)
N-St1-σ1.0	0.9276 (0.2558)	0.0499 (0.098)	0.8997 (0.2505)	0.3958 (0.2677)
N-St2-σ1.0	0.8351 (0.2947)	0.1874 (0.1448)	0.7772 (0.28)	0.4506 (0.2486)
N-All-σ1.0	0.9138 (0.2529)	0.1198 (0.1006)	0.8569 (0.2485)	0.4923 (0.2341)
N-Str-σ1.0	0.923 (0.2355)	0.1276 (0.1031)	0.885 (0.2299)	0.4258 (0.2338)
N-St1-σ1.5	0.8471 (0.3577)	0.0408 (0.0914)	0.8134 (0.3472)	0.2947 (0.2517)
N-St2-σ1.5	0.7821 (0.3447)	0.1517 (0.1301)	0.717 (0.3266)	0.3811 (0.2485)
N-All-σ1.5	0.8425 (0.3321)	0.0974 (0.0914)	0.783 (0.3179)	0.3838 (0.2479)
N-Str-σ1.5	0.8633 (0.309)	0.1039 (0.0944)	0.8112 (0.3033)	0.3414 (0.2312)
P-St1-σ0.5	0.9989 (0.0018)	0.0686 (0.1136)	0.9779 (0.0292)	0.6471 (0.2329)
P-St2-σ0.5	0.9379 (0.0683)	0.2952 (0.1595)	0.8817 (0.0897)	0.607 (0.2073)
P-All-σ0.5	0.9899 (0.0141)	0.1794 (0.1103)	0.9499 (0.0593)	0.7121 (0.1689)
P-Str-σ0.5	0.9885 (0.0156)	0.1972 (0.1141)	0.9609 (0.0385)	0.6241 (0.1922)
P-St1-σ1.0	0.9274 (0.2558)	0.0482 (0.0944)	0.8963 (0.2507)	0.388 (0.2619)
P-St2-σ1.0	0.8599 (0.2448)	0.2043 (0.1523)	0.7985 (0.2415)	0.4636 (0.2495)
P-All-σ1.0	0.9259 (0.2203)	0.1267 (0.1033)	0.8606 (0.2268)	0.4914 (0.2293)
P-Str-σ1.0	0.9358 (0.197)	0.1362 (0.1054)	0.8923 (0.1987)	0.4299 (0.2281)
P-St1-σ1.5	0.8171 (0.3848)	0.0402 (0.0892)	0.7817 (0.3719)	0.2931 (0.2536)
P-St2-σ1.5	0.7728 (0.348)	0.164 (0.1367)	0.7107 (0.3269)	0.3838 (0.2466)
P-All-σ1.5	0.803 (0.3685)	0.1011 (0.0929)	0.7461 (0.3499)	0.3813 (0.2473)
P-Str-σ1.5	0.8375 (0.3315)	0.1109 (0.0969)	0.7856 (0.3197)	0.3421 (0.2285)
S-St1-σ0.5	0.9974 (0.0084)	0.0537 (0.1832)	0.9825 (0.024)	0.2598 (0.3427)
S-St2-σ0.5	0.9237 (0.0358)	0.3677 (0.1456)	0.8655 (0.0413)	0.69 (0.1224)
S-All-σ0.5	0.9448 (0.0255)	0.338 (0.1417)	0.8901 (0.0309)	0.709 (0.1078)
S-Str-σ0.5	0.945 (0.0256)	0.338 (0.1417)	0.8975 (0.031)	0.658 (0.1181)
S-St1-σ1.0	0.8374 (0.3675)	0.0158 (0.0809)	0.8209 (0.3627)	0.1107 (0.2359)
S-St2-σ1.0	0.8578 (0.0844)	0.2596 (0.1421)	0.7662 (0.0922)	0.4767 (0.1599)
S-All-σ1.0	0.8849 (0.0775)	0.2323 (0.1345)	0.7847 (0.0871)	0.4892 (0.1541)
S-Str-σ1.0	0.8849 (0.0777)	0.2371 (0.1327)	0.8052 (0.0829)	0.4452 (0.1525)
S-St1-σ1.5	0.5978 (0.4908)	0.01 (0.0704)	0.5895 (0.4852)	0.0442 (0.1626)
S-St2-σ1.5	0.7676 (0.2549)	0.1719 (0.1476)	0.6765 (0.2318)	0.3146 (0.2055)
S-All-σ1.5	0.7862 (0.2527)	0.1488 (0.1315)	0.6766 (0.2331)	0.3104 (0.199)
S-Str-σ1.5	0.8094 (0.2181)	0.1577 (0.1352)	0.7261 (0.2041)	0.2894 (0.1903)

Table S4.I. The estimated FDR is 0.5 using the method: fdrtool

	H1		H1-Corr	
	FDR	sensitivity	FDR	sensitivity
N-St1-σ0.5	0.9994 (7e-04)	0.122 (0.1398)	0.9902 (0.0147)	0.8348 (0.1589)
N-St2-σ0.5	0.9722 (0.0356)	0.3338 (0.1716)	0.9333 (0.0679)	0.7348 (0.1787)
N-All-σ0.5	0.9968 (0.0028)	0.224 (0.1363)	0.98 (0.0287)	0.8608 (0.11)
N-Str-σ0.5	0.9964 (0.0031)	0.2431 (0.1372)	0.9833 (0.0202)	0.7779 (0.1423)
N-St1-σ1.0	0.9486 (0.2187)	0.0597 (0.1178)	0.9242 (0.2157)	0.538 (0.2792)
N-St2-σ1.0	0.8883 (0.2533)	0.2251 (0.1657)	0.8299 (0.2545)	0.5598 (0.2748)
N-All-σ1.0	0.9393 (0.2175)	0.135 (0.1124)	0.893 (0.2201)	0.6147 (0.2545)
N-Str-σ1.0	0.9498 (0.1953)	0.1538 (0.1193)	0.9162 (0.1938)	0.5511 (0.2542)
N-St1-σ1.5	0.8466 (0.3578)	0.0496 (0.1015)	0.8217 (0.3495)	0.4025 (0.295)
N-St2-σ1.5	0.8475 (0.3062)	0.1733 (0.1433)	0.7853 (0.293)	0.4803 (0.2611)
N-All-σ1.5	0.8552 (0.3337)	0.108 (0.0977)	0.8074 (0.3222)	0.481 (0.2884)
N-Str-σ1.5	0.8959 (0.2839)	0.1205 (0.1063)	0.8482 (0.279)	0.4435 (0.2543)
P-St1-σ0.5	0.9994 (8e-04)	0.1048 (0.1374)	0.9879 (0.0186)	0.8091 (0.1706)
P-St2-σ0.5	0.9647 (0.0394)	0.376 (0.1802)	0.9245 (0.0839)	0.7354 (0.1769)
P-All-σ0.5	0.9956 (0.004)	0.2326 (0.1367)	0.9748 (0.0387)	0.8453 (0.1176)
P-Str-σ0.5	0.995 (0.004)	0.2587 (0.1373)	0.9793 (0.0269)	0.7664 (0.1494)
P-St1-σ1.0	0.9586 (0.1967)	0.0543 (0.1091)	0.9273 (0.2033)	0.514 (0.2682)
P-St2-σ1.0	0.8969 (0.222)	0.2419 (0.1634)	0.8313 (0.2309)	0.5746 (0.2642)
P-All-σ1.0	0.953 (0.1765)	0.1394 (0.1113)	0.9055 (0.1822)	0.5995 (0.2488)
P-Str-σ1.0	0.9642 (0.1418)	0.1608 (0.1147)	0.9228 (0.1567)	0.5492 (0.2432)
P-St1-σ1.5	0.8564 (0.3477)	0.0473 (0.0981)	0.8324 (0.3401)	0.3869 (0.292)
P-St2-σ1.5	0.8536 (0.2928)	0.186 (0.1463)	0.7938 (0.2793)	0.4878 (0.2559)
P-All-σ1.5	0.865 (0.3223)	0.1116 (0.1)	0.8171 (0.3123)	0.4714 (0.2881)
P-Str-σ1.5	0.9119 (0.2536)	0.1265 (0.1034)	0.8668 (0.2483)	0.4416 (0.2499)
S-St1-σ0.5	0.9974 (0.0076)	0.0653 (0.1979)	0.9851 (0.0188)	0.3009 (0.3634)
S-St2-σ0.5	0.9391 (0.0257)	0.3961 (0.1416)	0.8919 (0.0313)	0.7366 (0.1108)
S-All-σ0.5	0.9561 (0.0181)	0.3648 (0.1372)	0.9126 (0.0238)	0.754 (0.1041)
S-Str-σ0.5	0.9563 (0.0182)	0.3648 (0.1372)	0.9182 (0.0232)	0.703 (0.1072)
S-St1-σ1.0	0.9183 (0.2723)	0.0158 (0.0809)	0.9049 (0.2697)	0.1518 (0.2725)
S-St2-σ1.0	0.9133 (0.0482)	0.2974 (0.145)	0.8398 (0.0576)	0.5937 (0.1549)
S-All-σ1.0	0.9341 (0.0362)	0.2659 (0.1403)	0.8535 (0.0557)	0.6089 (0.1426)
S-Str-σ1.0	0.9336 (0.0398)	0.2716 (0.1363)	0.8726 (0.0484)	0.5568 (0.1481)
S-St1-σ1.5	0.6985 (0.4597)	0.014 (0.0804)	0.6901 (0.4546)	0.091 (0.2271)
S-St2-σ1.5	0.8608 (0.1871)	0.1929 (0.151)	0.7657 (0.178)	0.4213 (0.2196)
S-All-σ1.5	0.878 (0.1965)	0.1737 (0.1409)	0.7737 (0.1898)	0.4266 (0.2195)
S-Str-σ1.5	0.9013 (0.1335)	0.1776 (0.1382)	0.8166 (0.1388)	0.3917 (0.2044)
